# RSK1 and RSK2 modulate the translatome of glioblastoma cells in an isoform-specific and mTORC1 independent manner

**DOI:** 10.1093/noajnl/vdaf144

**Published:** 2025-07-11

**Authors:** Martín Roffé, Danielle P Nascimento, Paula B Nunes, Luana C Soares, Arielly D H Alves, Ali Hamraghani, Yeganeh Almasi, Zakia Djaoud, Glaucia N M Hajj, Vilma R Martins, Nahum Sonenberg, Tommy Alain

**Affiliations:** National Institute of Science and Technology in Oncogenomics and Therapeutic Innovation (INCiTO), São Paulo, SP, Brazil; Children’s Hospital of Eastern Ontario Research Institute, Ottawa, ON, Canada; International Research Center (CIPE), A.C.Camargo Cancer Center, São Paulo, SP, Brazil; International Research Center (CIPE), A.C.Camargo Cancer Center, São Paulo, SP, Brazil; Department of Physiology, Anatomy and Genetics (DPAG), University of Oxford, Oxford, UK; Hospital Israelita Albert Einstein, São Paulo, SP, Brazil; Children’s Hospital of Eastern Ontario Research Institute, Ottawa, ON, Canada; Department of Biochemistry Microbiology and Immunology, University of Ottawa, Ottawa, ON, Canada; Children’s Hospital of Eastern Ontario Research Institute, Ottawa, ON, Canada; Department of Biochemistry Microbiology and Immunology, University of Ottawa, Ottawa, ON, Canada; International Research Center (CIPE), A.C.Camargo Cancer Center, São Paulo, SP, Brazil; National Institute of Science and Technology in Oncogenomics and Therapeutic Innovation (INCiTO), São Paulo, SP, Brazil; Department of Cell Biology and Development, Institute of Biomedical Sciences, University of São Paulo, São Paulo, SP, Brazil; International Research Center (CIPE), A.C.Camargo Cancer Center, São Paulo, SP, Brazil; National Institute of Science and Technology in Oncogenomics and Therapeutic Innovation (INCiTO), São Paulo, SP, Brazil; Department of Biochemistry, McGill University, Montreal, QC, Canada; Rosalind and Morris Goodman Cancer Institute, McGill University, Montreal, QC, Canada; Department of Biochemistry Microbiology and Immunology, University of Ottawa, Ottawa, ON, Canada; Children’s Hospital of Eastern Ontario Research Institute, Ottawa, ON, Canada

**Keywords:** RSK, mTORC1, glioblastoma, signaling, mRNA translation

## Abstract

**Background:**

The p90 ribosomal S6 kinase (RSK) family, downstream target of Ras/ERK signaling, encompasses four human isoforms (RSK1-4). Glioblastomas (GBMs) predominantly express RSK1 and RSK2, whereby RSK1 is markedly upregulated in a subset of GBMs associated with dismal prognosis and immune infiltration, while RSK2 expression is constant. RSKs were proposed as regulators of mRNA translation through the activation of mTORC1 and other factors, such as eIF4B, but nothing is known about their effect on the translatome of GBM cells.

**Methods:**

Through the generation of RSK1 and RSK2 knockout as well as double knockout (DKO) GBM cells, we investigated RSK isoform-specific functions in cell signaling, followed by the identification of their distinct transcriptome and translatome.

**Results:**

We find that mTORC1 is not activated by RSK isoforms and that eIF4B phosphorylation at S422 is more potently targeted by RSK1 than mTORC1/S6K in GBM cells. Intriguingly, RSK isoforms display differential effects on translation, with RSK1 specifically sustaining translation of a subset of mRNAs upon mTORC1 inhibition. We demonstrate that RSK1 modulates expression in the translatome of mRNAs encoding proteins affecting cell cycle, DNA replication, and repair, while RSK2 impacts mitochondria-related functions. Notably, DKO cells exhibit compounded phenotypes, underscoring the existence of isoform-specific gene regulation.

**Conclusions:**

Our findings offer mechanistic insights into the role of RSK in GBMs and provide evidence for a mTORC1-independent and RSK1-dependent translation regulatory program.

**Key Points**

- RSK isoforms regulate mRNA translation in a mTORC1-independent manner in glioblastoma cells.
- RSK1 and RSK2 distinctively modulate the translatome of glioblastoma cells.
- RSK1 sustains translation of mRNAs upon mTORC1 inhibition in glioblastoma cells.

**Importance of the Study**

We previously identified a subgroup of glioblastomas (GBMs) characterized by high expression levels of RSK1, one of the four RSK isoforms in humans. Interestingly, this subgroup was associated with poor survival and increased immune cell infiltration. Here, we demonstrate that GBM cell lines recapitulate the expression pattern of RSK isoforms. Although RSKs were identified as regulators of mRNA translation, nothing is known about their effects on the translatome in GBMs. We show that RSK-mediated regulation of mRNA translation in GBM cells occurs independently of mTORC1. Using CRISPR/Cas9, we generated knockout cells for the two isoforms expressed in GBM, RSK1, and RSK2, along with double-knockout cells. With these cells, we identified the isoform-specific translatome and defined distinct gene-expression programs, with RSK1 linked to cell cycle and DNA repair regulation, and RSK2 to mitochondrial functions. Finally, we uncover an RSK1-dependent translation program that is activated upon treatment with an mTOR inhibitor.

Gliomas are central nervous system tumors that result from the malignant transformation of glial cells, their intermediate precursors, or neural stem cells.^1,2^ The most malignant type of glioma is glioblastoma (GBM). According to the 2021 World Health Organization (WHO) classification, GBMs are grade 4 tumors and do not exhibit mutations in the isocitrate dehydrogenase (IDH) genes.^3^ GBM is highly lethal, with patients typically surviving only 12–15 months.^4^ Standard treatment involves surgical resection, when possible, but due to its infiltrative nature, complete removal of GBMs is not achievable. Therefore, adjuvant treatment, including radiotherapy and chemotherapy with the alkylating agent temozolomide, is administered after surgery.^5^ However, almost all GBMs eventually recur, with a mean disease progression time of 6.9 months after treatment.^6^

The Ras/extracellular signal-regulated kinase (ERK) signaling pathway is frequently altered in GBMs.^7,8^ A direct target of ERK signaling is the p90 kDa ribosomal protein S6 kinase (RSK) family, which comprises four isoforms in humans (RSK1–4; Figure 1A).^9^ Previous analyses found that a subgroup of about 1/3 of GBMs expresses high levels of RSK1 compared to other GBMs, lower-grade gliomas (grades I-III; LGG), and non-tumor brain (NB).^10^ In contrast, the RSK2 isoform showed similar expression among GBMs, LGGs, and NB, and the other two isoforms, RSK3 and RSK4, were either barely detected or not detected, respectively.^10^ Interestingly, aberrantly high RSK1 levels were observed upon recurrence and correlated with poor survival and immune infiltration in GBMs.^10^ RSK isoforms are highly homologous, sharing 75–80% amino acid identity,^9^ yet no clear isoform-specific mechanisms of action have been described in glioblastoma (GBM). Furhermore, the determinants of the proposed distinct functions of RSK1 and RSK2 remain poorly understood. However, the fact that RSK inhibitors, such as PMD-026, are currently being tested in clinical trials,^11^ underscores the importance of the RSK family as emerging targets for cancer treatment.

**Figure 1.**
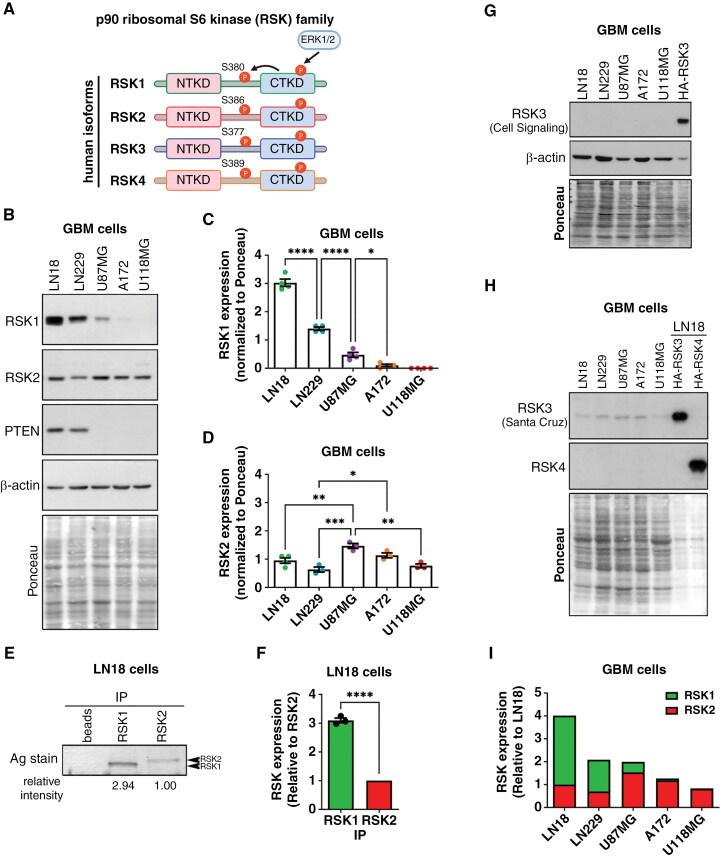
**Protein expression levels of the RSK isoforms in GBM cells.** (**A**) Scheme of the four human isoforms of the p90 ribosomal S6 kinase (RSK) family. After ERK1/2 phosphorylation of the C-terminal kinase domain (CTKD), RSK isoforms are auto-phosphorylated by the CTKD, which is necessary for the complete activation of the N-terminal kinase domain (NTKD). Created in BioRender. Roffe, M. (2025) https://BioRender.com/ic8gw5j. (**B**) Western blot was used to determine the protein levels of RSK1, RSK2, and PTEN in the indicated GBM cell lines. Western blot for β-actin and the Ponceau-stained membrane were used as loading controls. (**C, D**) Quantification of (**C**) RSK1 and (**D**) RSK2 protein levels in western blots. The intensity values were normalized by the amount of protein in the corresponding Ponceau-stained lane. The mean values of four independent experiments (± SEM) are presented. (**E**) Immunoprecipitated RSK1 and RSK2 were separated on a polyacrylamide gel and silver (Ag)-stained. RSK1 to RSK2 relative expression was estimated by quantifying band intensities in the silver-stained gel, with values corrected for IP efficiency (Supplementary Figure S3). RSK1/RSK2 relative intensity is shown below the panel. (**F**) Graph showing the RSK1/RSK2 relative content in LN18 cells (Figure 1E), after correction for IP efficiency. Mean values of three independent experiments (± SEM) are presented. (**G**) Western blot using a *Cell Signaling* antibody was performed to determine the protein levels of RSK3 in the indicated GBM cell lines. As a positive control, we included extracts obtained from LN18 cells that were transiently transfected to express HA-RSK3. Three times less extract of HA-RSK3 expressing cells was used. The western blot for RSK3 was over-exposed. Western blot for β-actin and the Ponceau-stained membrane were used as loading controls. (**H**) Western blot was used to determine the protein levels of RSK3 and RSK4 in the indicated GBM cell lines. For RSK3, a *Santa Cruz* antibody was used, and the western blots were over-exposed. As positive controls, we included extracts obtained from LN18 cells that were transiently transfected to express HA-RSK3 or HA-RSK4. Three times less extract of transfected cells was used. The Ponceau-stained membrane was used as a loading control. (**I**) Graph showing estimation of RSK1/RSK2 relative content for GBM cell lines.

Upon activation, RSKs phosphorylate several targets in the cytosol and nucleus, contributing to various cellular functions, including cell proliferation, growth, survival, and migration.^12^ Additionally, RSKs are proposed to mediate cross-talk with the mechanistic Target of Rapamycin complex I (mTORC1) signaling pathway (scheme in Supplementary Figure S1A). This is significant because most GBMs show alterations in at least one of the major components of the receptor tyrosine kinase/phosphatidylinositol-3 kinase/protein kinase B signaling (RTK/PI3K/AKT) pathway, which is upstream of mTORC1.^7,8,13^ The cross-talk is proposed to occur through the direct phosphorylation of tuberous sclerosis complex 2 (TSC2)^14^ and regulatory associated protein of mTOR (Raptor)^15^ by RSKs, leading to mTORC1 activation. Upon activation, mTORC1 phosphorylates its direct targets, including eukaryotic translation initiation factor 4E binding proteins (4E-BPs) and p70 kDa ribosomal protein S6 kinase (S6K).^9^ However, it was previously observed that a reduction in TSC2 phosphorylation upon RSK depletion did not result in the expected inactivation of mTORC1 in GBM cell lines, raising questions about the association of this phosphorylation with mTORC1 activation.^16^ Additionally, RSKs can mediate cross-talk with mTORC1 signaling by phosphorylating the same residues of S6K substrates, including ribosomal protein S6 (RPS6),^17^ eukaryotic translation initiation factor 4B (eIF4B),^18^ and eukaryotic translation elongation factor 2 kinase (eEF2K).^19^ However, it is noteworthy that the elucidation of the relevance of this cross-talk was obscured by the widespread use of RSK inhibitors that show nonspecific effects on the mTORC1 signaling pathway.^16^

Translation is a major output for the regulation of gene expression, and mTORC1, through the phosphorylation of its targets, is one of its most important regulators.^20^ Therefore, RSKs may regulate translation indirectly through cross-talk with mTORC1 or directly by phosphorylating translation-regulatory proteins, including those targeted by S6K. In this way, RSKs could function as modulators of the translatome, which is the fraction of the transcriptome actively translated to shape the proteome of cells.^21^ Despite the potential role of RSKs as regulators of the translation process, no translatomic studies have been conducted, and little is known about the isoform-specific regulation of translation by RSKs, particularly in GBMs.

Here, we demonstrate that GBM-derived cell lines recapitulate the RSK isoform expression characteristics of GBM tissues, and we define RSK isoform-specific gene expression programs at the translatome level.

## Materials and Methods

See Supplementary Materials and Methods for detailed experimental procedures.

### Cell Culture

LN18 (ATCC^®^ CRL-2610™), LN229 (ATCC^®^ CRL-2611™), U87MG (ATCC^®^ HTB-14™), A172 (ATCC^®^ CRL-1620™), and U118MG (ATCC^®^ HTB-15™) cells were cultured in Dulbecco’s Modified Eagle Medium (DMEM), high glucose, GlutaMAX, pyruvate (Gibco^®^), supplemented with 10% fetal calf serum. LN18 cells were transfected to express influenza hemagglutinin (HA)-tagged RSK3 (HA-RSK3) or RSK4 (HA-RSK4) using Lipofectamine^®^ 2000 (Invitrogen™). These plasmids were described previously.^22^

### Generation of RSK-knockout GBM Cells Using CRISPR/Cas9

The CRISPR/Cas9 system was employed to edit the genome of LN18 and LN229 cells following the protocol published by Ran et al.^23^ with minor modifications. Briefly, sgRNAs targeting *RSK1* (*RPS6AK1*) and *RSK2* (*RPS6KA3*) genes were designed using the guide design tool at https://portals.broadinstitute.org/gppx/crispick/public. The DNA oligonucleotides encoding the sgRNAs (see Supplementary Table S2) were cloned into the pSpCas9(BB)-2A-Puro (PX459) V2.0 plasmid (Addgene), which encodes the wild-type spCas9 and a puromycin resistance cassette. To generate double knockout (DKO) cells, cells were transfected with two plasmids for sgRNA targeting either RSK1 (sgRSK1) or RSK2 (sgRSK2). Clones of cells transfected with an empty plasmid lacking sgRNAs were used as controls (WT). Individual clones were expanded, and the expression of RSK1 and RSK2 was assessed by Western blotting.

### Non-linear Polysome Profiling

Cytosolic extracts were applied to a sucrose non-linear gradient made in 20 mM Tris-HCl pH 7.5, 100 mM NaCl, 5 mM MgCl_2_, and 1 mM DTT, as previously described.^24^ The gradients were centrifuged at 35,000 RPM for 2 h at 4 °C in a SW41Ti rotor and Optima XE-90 Ultracentrifuge (Beckman Coulter). Translation levels of LN18^CRISPR^ cells were estimated by the ratio of the area under the peak of efficiently translated mRNAs to the area under the peaks of poorly translated mRNAs and monosomes (Figure 3C, D).

### Microarray Assay

RNA was purified from the fractions of the non-linear gradient containing polysome-associated mRNA and from an aliquot of the original cytosolic extracts (total mRNA) using TRI-Reagent^®^ (Sigma Aldrich) and the Direct-zol^TM^ RNA MiniPrep kit (Zymo Research). The integrity of the RNA was evaluated using the RNA 6000 Pico Chips kit in a 2100 Bioanalyzer (Agilent Technologies). Both total and polysome-associated mRNA were subjected to the Clariom^TM^ D human microarray (Thermo Fisher Scientific) according to the manufacturer’s instructions.

### Gene Expression Analysis

Normalized polysome-associated and total mRNA were analyzed by the anota2seq package.^25^ Anota2seqSelSigGenes filter parameters were set as follows: maxSlopeTranslation = 2; minSlopeTranslation = − 1; maxSlopeBuffering = 1; minSlopeBuffering = − 2; deltaPT = log2(1.2); deltaTP = log2(1.2); deltaP = log2(1.5); deltaT = log2(1.5), maxP = 0.05.

### Statistical Analysis

One-way ANOVA followed by Tukey’s post hoc test or Student’s *t*-test was applied after assuming normality. *P* < .05 were considered statistically significant (* or ^#^). Pearson’s and Spearman’s coefficients for correlations are indicated in the graphs alongside with the *P-values* in parentheses. Statistical analysis and graphs were performed using GraphPad Prism or RSTUDIO with R version 4.2.1.

## Results

### Expression of RSK Isoforms in GBM Cell Lines

We previously investigated the protein levels of each of the four human RSK isoforms (Figure 1A) in tissues derived from GBMs.^10^ To confirm whether these observations are replicated in GBM cells in culture, we analyzed the levels of RSK1-4 in five GBM cell lines (LN18, LN229, U87MG, A172, and U118MG) using western blotting. RSK1 protein expression varied significantly, with each cell line expressing different levels (Figure 1B, C). LN18 cells displayed the highest protein levels, followed by LN229 cells. Conversely, RSK1 was barely detected or not detected at all in A172 and U118MG cells, respectively. RSK1 showed a similar expression pattern at both the mRNA and protein levels, indicating a high correlation between the two (Supplementary Figure S2A, B), consistent with observations in patient-derived GBMs.^10^ Intriguingly, the two cell lines with the highest levels of RSK1 harbor wild-type PTEN^26^ (Figure 1B). In contrast, RSK2 protein expression was more uniform among the cell lines, with all cells expressing detectable levels (Figure 1B, D and Supplementary Figure S2C, D). To assess the stoichiometry of RSK1 and RSK2 isoforms, we quantitatively immunoprecipitated RSK1 and RSK2 from LN18 cells, which express the highest levels of RSK1, and compared their relative expression (Figure 1E and Supplementary Figure S3). We found that the levels of RSK1 were approximately three times higher than the levels of RSK2 in LN18 cells (Figure 1F).

Our analyses revealed that RSK3 is either expressed at very low levels or not at all in the five cell lines tested, and that RSK4 protein was undetectable (Figure 1G, H and Supplementary Figure S4). However, RSK3 mRNA was detected in all five cell lines and RSK4 mRNA was only detected in two of them (Supplementary Figure S2E, F). Given that RSK3 and RSK4 contribute minimally, if at all, to total RSK isoform levels in GBM cells, we estimated overall RSK abundance based on RSK1 and RSK2 expression and stoichiometry (Figure 1I and Supplementary Figure S3A). These data show that while the predominant RSK isoform in LN18 and LN229 cells is RSK1, RSK2 is the predominant isoform in U87MG and A172 cells; U118MG cells only express RSK2. In summary, the expression pattern of RSK isoforms in GBM cell lines recapitulates that observed in patient-derived GBM tissue,^10^ with RSK1 and RSK2 being the two main isoforms expressed by these tumors.

### Isoform-specific Regulation of RSK Signaling in GBM Cells

**Figure 2.**
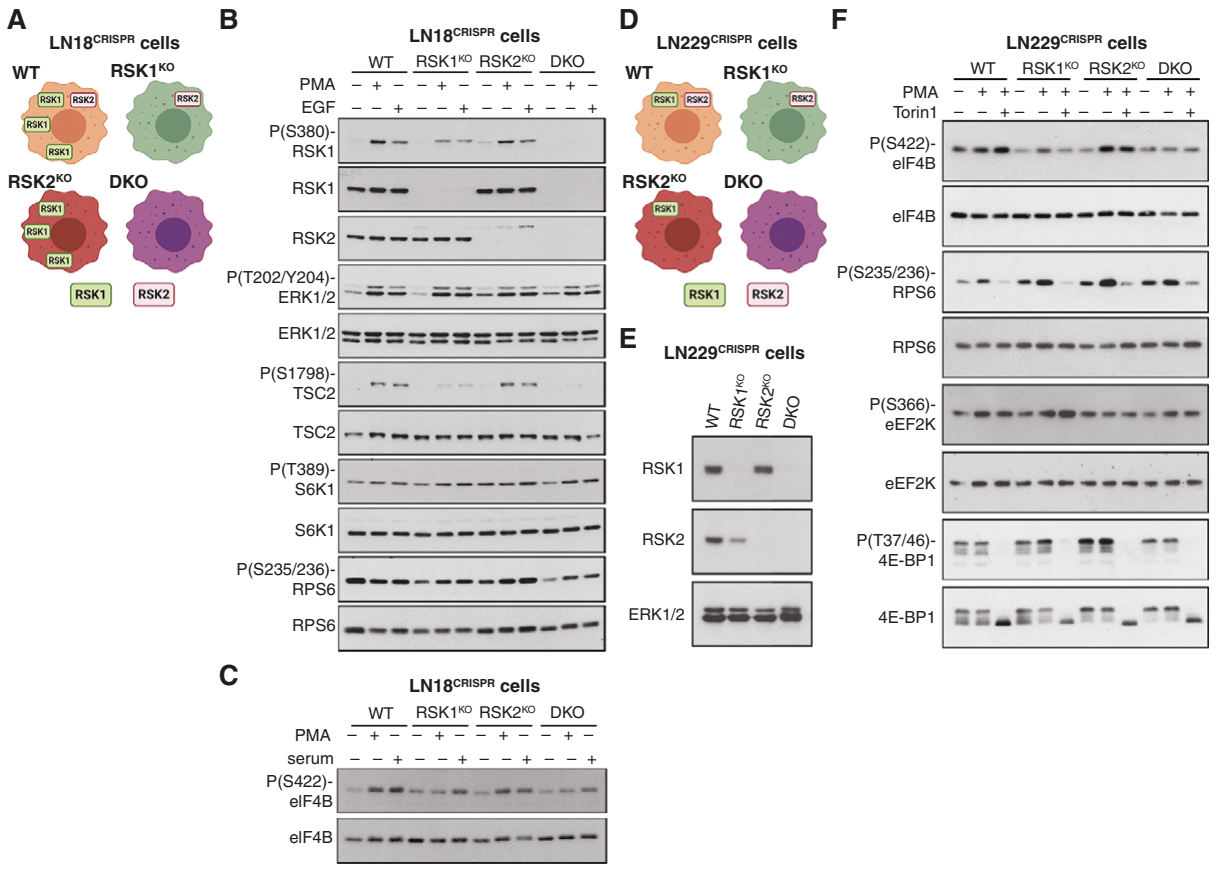
**RSK isoform-specific cross-talk with mTORC1/S6K signaling in GBM cells.** (**A**) Illustration showing the RSK-isoform content of LN18 cells edited by CRISPR/Cas9 (LN18^CRISPR^ cells). Created in BioRender. Roffe, M. (2025) https://BioRender.com/zaq47hh. (**B**) LN18^CRISPR^ cells were serum-starved for 48 h and then treated with either PMA or EGF for 15 min. Western blots were performed for the indicated proteins and its phosphorylated forms. (**C**) LN18^CRISPR^ cells were serum-starved for 48 h and then treated with PMA or serum for 15 min. Western blots were performed for the indicated total and its phosphorylated forms. (**D**) Illustration showing the RSK-isoform content of LN229 cells edited by CRISPR/Cas9 (LN229^CRISPR^ cells). Created in BioRender. Roffe, M. (2025) https://BioRender.com/0rv2wb8. (**E**) Western blot of LN229^CRISPR^ cells was performed to detect RSK1 and RSK2 protein expression. Western blot for ERK1/2 was used as loading control. (**F**) LN229^CRISPR^ cells were serum-starved for 48 h and then treated with PMA for 15 min in the absence or presence of 250 nM Torin1. Western blots were performed for the indicated proteins and their phosphorylated forms. Graphs showing the quantifications can be found in Supplementary Figure S6.

The heterogeneity of RSK1 expression in both patient-derived GBMs and GBM cell lines might suggest the existence of isoform-specific roles in this tumor type. To define RSK-isoform-specific functions, we generated knockout LN18 (LN18^CRISPR^) and LN229 (LN229^CRISPR^) cells for RSK1 (RSK1^KO^) or RSK2 (RSK2^KO^) using CRISPR/Cas9 as well as double-knockout cells for RSK1 and RSK2 (DKO) (Figure 2A, H). By western blot, we confirmed the absence of RSK1 and/or RSK2 in the corresponding CRISPR-targeted cells (Figure 2B, E). A slight reduction in viability was observed in the three LN18^CRISPR^ knockout cell lines (Supplementary Figure S5A). However, only RSK1^KO^ and DKO LN229^CRISPR^ cells exhibited a decrease in viability in growth medium, suggesting an isoform-specific dependence on RSK1 in this cell line (Supplementary Figure S5B).

Focusing first on the LN18^CRISPR^ cells, we examined the effects of RSK isoforms on known signaling substrates common to the mTORC1/S6K signaling pathway. After serum starvation, cells were treated with either PMA or EGF to activate ERK/RSK signaling, resulting in an expected increase in ERK1/2 phosphorylation (Figure 2B). No apparent differences regarding ERK1/2 phosphorylation were observed between wild-type and knockout LN18^CRISPR^ cells, suggesting that at least in these cells and conditions, negative feedback of ERK signaling^12,27,28^ was not clearly affected by RSK isoform loss (Supplementary Figure S6A). Moreover, the knockout cells allowed us to verify that the P(S380)-RSK1 antibody detects both P(S380)-RSK1 and P(S386)-RSK2 (Figure 2B). Phosphorylation at S1798 of TSC2, a direct RSK substrate, was completely abolished in DKO cells (Figure 2B and Supplementary Figure S6B). It was proposed that TSC2 phosphorylation by RSKs results in mTORC1 activation^14^; however, we did not observe that reduction in TSC2 phosphorylation results in a concomitant reduction in S6K1 phosphorylation at T389, which is mediated by mTORC1 (Figure 2B and Supplementary Figure S6C). Thus, we confirmed that TSC2 phosphorylation at S1798 is not related to mTORC1 activity in GBM cells.^16^ RPS6 phosphorylation at S235/236 can be mediated by both S6K and RSK (Supplementary Figure S1A). However, only DKO cells showed reduced levels of RPS6 phosphorylation, indicating that in LN18^CRISPR^ cells, these residues are mainly phosphorylated by S6K and that little contribution of RSK is noted only in the absence of all RSK isoforms (Figure 2B and Supplementary Figure S6D). Another shared target between RSK and mTORC1/S6K is S422 on eIF4B.^18^ The PMA-induced increase of eIF4B phosphorylation at S422 was significantly reduced in DKO cells, indicating that this phosphorylation is dependent on RSK rather than on mTORC1/S6K in LN18^CRISPR^ cells (Figure 2C and Supplementary Figure S6E). Furthermore, RSK1^KO^ cells showed reduced levels of PMA-induced eIF4B phosphorylation, while levels in RSK2^KO^ cells were unaffected, indicating an isoform-specific preference.

To better understand the isoform-specific effects of RSK, we utilized LN229^CRISPR^ cells (Figure 2D, E). We noted a slight reduction in RSK2 levels in RSK1^KO^ LN229^CRISPR^ cells, which was consistent across different clones (Supplementary Figure S7). Similar to S6K phosphorylation in LN18^CRISPR^ cells, loss of RSK isoforms in LN229^CRISPR^ cells did not influence mTORC1 activity toward 4E-BP1 phosphorylation levels, further confirming that mTORC1 is not regulated by RSKs in GBM cells (Figure 2J and Supplementary Figure S6F). RSK1^KO^ LN229^CRISPR^ cells also showed impaired induction of eIF4B phosphorylation at S422, while this phosphorylation was not altered in RSK2^KO^ cells (Figure 2J and Supplementary Figure S6G). To elucidate the possible role of mTORC1 in the effects of RSK, LN229^CRISPR^ cells were pre-treated with the mTOR inhibitor, Torin1,^29^ before stimulation with PMA. PMA-induced phosphorylation of eIF4B in LN229 cells was unaffected by Torin1 treatment, indicating that it is not dependent on mTORC1. These results indicate that S422 phosphorylation of eIF4B is preferentially mediated by RSK1 rather than RSK2 in PMA-stimulated GBM cells that express RSK1. Torin1 treatment inhibited RPS6 phosphorylation under all conditions, consistent with this phosphorylation being mainly dependent on mTORC1/S6K in GBM cells (Figure 2J and Supplementary Figure S6H). Finally, phosphorylation of S366 on eEF2K, reported to be dependent on both S6K and RSK,^19^ was not reduced by the lack of RSK isoforms (Figure 2J and Supplementary Figure S6I). The basal phosphorylation levels were also not reduced by Torin1 in LN229^CRISPR^ cells. These observations suggest that eEF2K is not modulated by either mTORC1 or RSK isoforms in these GBM cells under the tested conditions. Altogether, our analysis of different RSK substrates showed varying responses upon RSK activation, indicating that other factors are also important for their regulation.

### RSK1 and RSK2 Isoforms Differentially Regulate the Translatome of LN18^CRISPR^ Cells

**Figure 3.**
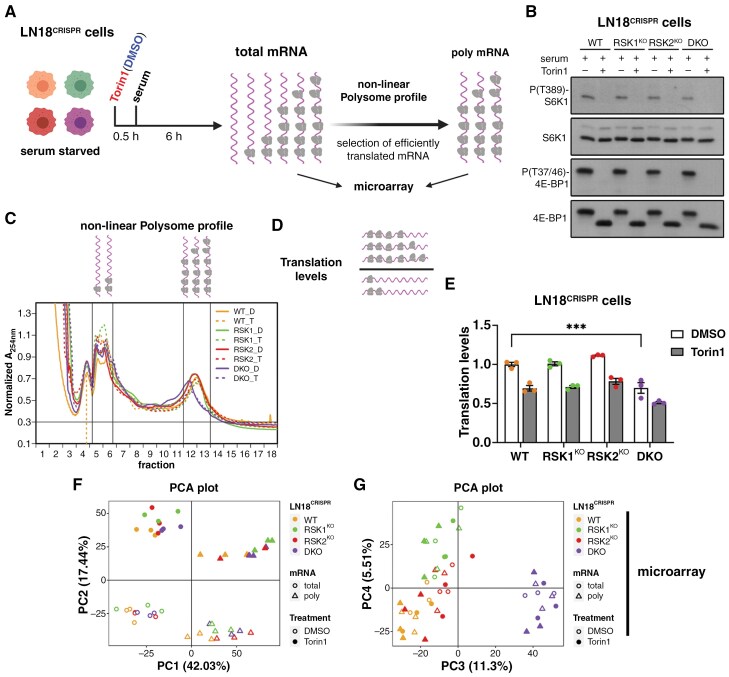
**RSK isoform effects on translation and overall gene expression.** (**A**) Scheme of the approach to assess the transcriptome and translatome of LN18^CRISPR^ cells. Created in BioRender. Roffe, M. (2025) https://BioRender.com/n76d8jx. (**B**) LN18^CRISPR^ cells were serum-starved for 48 h and then treated with serum for 6 h in the presence or absence of Torin1. Western blots were performed for the indicated proteins and its phosphorylated forms. (**C**) Non-linear polysome profiles made with cytosolic extracts of LN18^CRISPR^ cells that were treated as in (**B**). Above the graph, a scheme of the composition of the indicated peaks is shown. The fractions containing efficiently translated mRNAs were used to isolate polysome-associated mRNA. The area under the peaks that were quantified for the estimation of translation levels is delimited by black lines. (**D**) Scheme of the calculation of the translation levels of the LN18^CRISPR^ cells. Created in BioRender. Roffe, M. (2025) https://BioRender.com/y411dy7. (**E**) Graph showing the quantification of the translation levels of LN18^CRISPR^ cells. The mean values of three independent experiments (± SEM) are presented. ***P = .0002 (one-way ANOVA followed by Tukey’s post hoc test). (**F, G**) Total and polysome-associated (poly) mRNA obtained from LN18^CRISPR^ cells were analyzed by microarray, and the expression data were used to perform principal component analysis (PCA). (**F**) The plot shows PC1 and PC2. (**G**) The plot shows PC3 and PC4.

To investigate the role of RSK isoforms in gene expression, LN18^CRISPR^ cells were serum-starved for 48 h and then treated with serum for 6 h. To define RSK-isoform specific effects on the translatome that are dependent or independent of mTORC1 activity, cells were also pretreated with Torin1 before the pulse with serum (Figure 3A). As expected, we did not observe any difference in the phosphorylation state of mTORC1 downstream targets, S6K1 and 4E-BP1, consistent with a lack of RSK-mediated mTORC1 regulation in LN18^CRISPR^ cells under serum stimulation (Figure 3B). Furthermore, Torin1 pretreatment completely abolished S6K1 and 4E-BP1phosphorylation in all LN-18^CRISPR^ cells. Cytosolic extracts were obtained and used for transcriptomics and translatomics analyses. For translatome analysis, we utilized a modified polysome profiling technique using a non-linear gradient^24^ (Figure 3C). Translation levels in WT, RSK1^KO^, and RSK2^KO^ cells were comparable, and Torin1 reduced translation in all three cell lines to the same extent (Figure 3D, E). Strikingly, translation levels of DKO cells were reduced even in the absence of Torin1 treatment (Figure 3E). It is important to mention that since 4E-BP1 and S6K1 phosphorylation levels were not affected in DKO cells (Figure 3B), the translation impairment was not related to the inactivation of mTORC1.

Efficiently translated mRNA (mRNA in heavy polysomes), together with total mRNA directly purified from the cytosolic extracts, were analyzed by microarray (Figure 3A) and principal component analysis (PCA) plots were performed to get insights into the overall effects of RSK isoform deficiency on the transcriptome (total mRNA) and translatome (polysome-associated mRNA) (Figure 3F, G and Supplementary Figure S8A, B). We observed that mRNA source—translatome *vs* transcriptome—defines the principal component 1 (PC1), and treatment—DMSO versus Torin1—defines PC2 (Figure 3F). PC3 separated DKO from the remaining cells and PC4 demonstrated that RSK1 loss impacted gene expression more than RSK2 loss (Figure 3G). Altogether, these results indicate that RSKs are major regulators of gene expression in LN18 cells.

**Figure 4.**
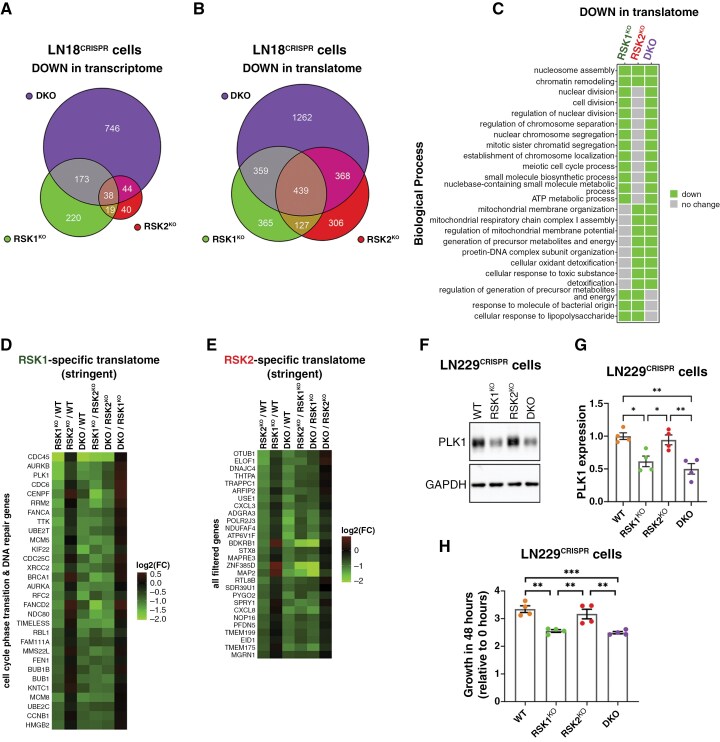
**RSK isoforms effects on the translatome.** (**A**) Total (transcriptome) and (**B**) Polysome-associated mRNA (translatome) from RSK1^KO^, RSK2^KO^, and DKO LN18^CRISPR^ cells were compared to the transcriptome and translatome, respectively, of WT LN18^CRISPR^ cells. The Venn diagram shows the number of downregulated mRNAs (*P* < .01) for each knockout cell line. (**C**) Downregulated biological processes in the translatome shared between knockout cells. (**D**) Polysome-associated mRNAs that were downregulated in all the comparisons where RSK1 is absent but not when RSK2 is absent were selected. The heatmap shows a fraction of those filtered mRNAs associated with cell cycle transition and DNA repair biological processes. (**E**) Polysome-associated mRNAs that were downregulated in all the comparisons where RSK2 is absent but not when RSK1 is absent were selected. All the filtered genes are included in the heatmap. (**F**) Western blot for PLK1 in LN229^CRISPR^ cells. GAPDH was used as a loading control. (**G**) Graph showing the quantification of PLK1 from western blots. The mean values of four independent experiments (± SEM) are presented. (**H**) LN229^CRISPR^ cell growth was monitored by measuring the confluence using the Incucyte live-cell imaging system. The ratio of the confluence between 48 h and 0 h is represented in the graph. The mean values of four independent experiments (± SEM) are presented.

While RSK1 depletion was associated with the downregulation of 450 mRNAs in the transcriptome, only 141 genes were downregulated in the transcriptome of RSK2^KO^ cells (Figure 4A). In the translatome, RSK1^KO^ and RSK2^KO^ cells showed a downregulation of a similar number of genes, with approximately 45% of these genes being shared between the two knockouts (Supplementary File S1 and Figure 4B). Additionally, more than 60% of genes downregulated in the translatome of RSK1^KO^ and RSK2^KO^ cells, respectively, were also downregulated in DKO cells (Figure 4B). The number of downregulated genes in the translatome of DKO cells was almost double than that observed in either individual knockout cells, indicating that the lack of both RSK isoforms has a profound impact on gene expression in GBM cells. To gain insight into the classes of genes that are dependent on RSK isoforms, we examined the related biological processes (BP) (Supplementary File S2). RSK1^KO^ cells were characterized by the downregulation of genes associated with cell division, replication, and DNA repair (Supplementary Figure S9A). In contrast, RSK2^KO^ cells showed an underrepresentation of mitochondrial- and energy-related BPs, which was not evident in RSK1^KO^ cells (Supplementary Figure S9B). Additionally, DKO cells showed underrepresentation of BPs that were also downregulated in RSK1^KO^ or RSK2^KO^ cells; however, most of these BPs were not shared between the two individual KO cells (Supplementary Figure S9C). For example, DKO cells shared the downregulation of cell division with RSK1^KO^ cells and mitochondrial-related BPs with RSK2^KO^ cells (Figure 4C). These findings suggest that RSK1 and RSK2 have independent gene regulation programs that are simultaneously impacted in DKO cells. Many of the BPs downregulated in the translatome of RSK1^KO^ cells are also observed in their transcriptome (Supplementary Figure S9D); however, in RSK2^KO^ cells, changes in BPs are essentially observed at the level of the translatome but not the transcriptome. Particularly, changes in mitochondrial-related BPs are not evident in the transcriptome of both RSK2^KO^ and DKO cells (Supplementary Figure S9E, F).

To classify genes modulated in the translatome by RSK1 but not RSK2, we selected the mRNAs downregulated in all of the following comparisons involving the absence of RSK1: RSK1^KO^/WT; DKO/WT; RSK1^KO^/RSK2^KO^; DKO/RSK2^KO^ (Supplementary File S3). Concordantly, we excluded the mRNAs that were downregulated in a RSK2-dependent manner: RSK2^KO^/WT; DKO/RSK1^KO^. We identified 126 RSK1-specific mRNAs, most of which were related to cell division, cell cycle phase transition, and DNA repair. These mRNAs include those coding for CDC45, TIMELESS, PLK1, and BRCA1 (Figure 4D). Applying the same analysis for RSK2^KO^ cells, only 33 RSK2-specific mRNAs were identified (Figure 4E and Supplementary File S3); however, no clear BP was associated with these fewer genes. Confirming the RSK1-specific dependence of PLK1 expression, its protein expression was also downregulated in RSK1^KO^ and DKO, but not in RSK2^KO^ LN229^CRISPR^ cells (Figure 4F, G). As expected, both RSK1^KO^ and DKO LN229^CRISPR^ cells exhibited reduced proliferation rates (Figure 4H and Supplementary Figure S10A). Despite the reduced viability observed in RSK1^KO^ and DKO LN18^CRISPR^ cells, they did not show detectable reduction in proliferation rates compared to WT cells under standard growth conditions (Supplementary Figure S10B). This apparent paradox was previously observed in RSK1^KO^ U251MG cells, which showed downregulation of genes involved in cell cycle and DNA replication, without measurable effects on proliferation or cell cycle progression.^30^ However, under serum starvation or after incubation with PMA, DKO LN18^CRISPR^ cells displayed reduced BrdU incorporation into DNA (Supplementary Figure S10C).

The variable expression of RSK1 in GBM cells was also evident in a quantitative proteomics analysis of GBM cells of the Cancer Cell Line Encyclopedia (CCLE) (Supplementary Figure S11A).^31^ The analysis of CCLE data revealed that almost all proteins encoded by RSK1-specific mRNAs showed a positive correlation with RSK1 in the 12 GBM cell lines (including the 5 GBM cell lines used here) of the CCLE, being FEN1 and MCM8 the highest correlated proteins (Supplementary File S3 and Supplementary Figure S11C, D). However, we did not observe the same for the RSK2-specific translatome (Supplementary Figure S11B and Supplementary File S3). These findings suggest that RSK1 might have a pivotal role in gene expression of GBM cell lines expressing high levels of RSK1.

### Effects of RSK1 and RSK2 on Translation Efficiency in LN18^CRISPR^ Cells

**Figure 5.**
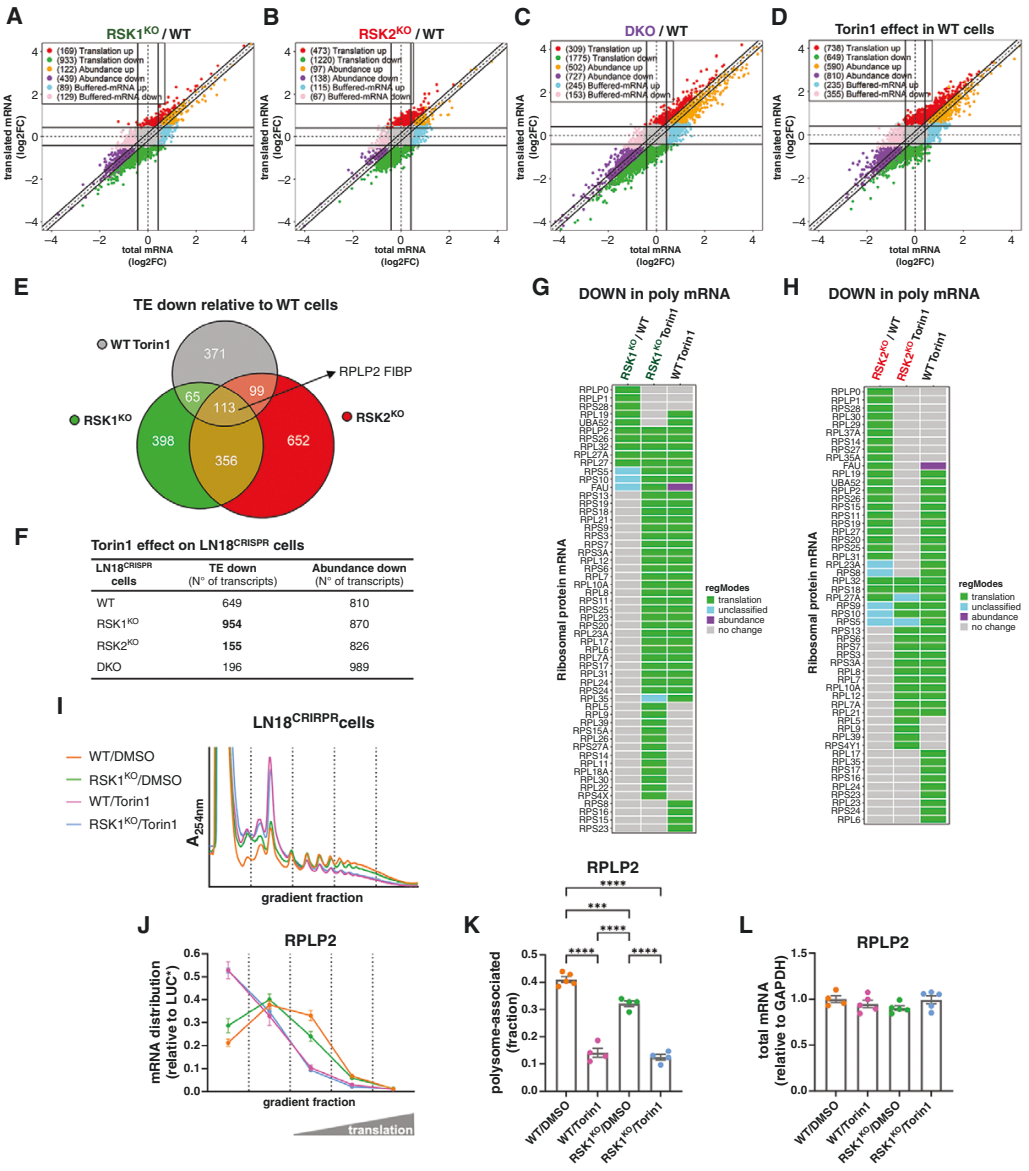
**Effect of RSK isoforms on translation efficiency (TE).** (**A-C**) Graphs showing changes in gene expression and the regulatory modes assigned according to the anota2seq method for the contrasts (**A**) RSK1^KO^/WT, (**B**) RSK2^KO^/WT, and (**C**) DKO/WT. The number of genes classified in each mode is indicated within parentheses. (**D**) Graph showing changes in gene expression and the regulatory modes assigned according to the anota2seq method for Torin1-treated WT cells. The number of genes classified in each mode is indicated within parentheses. (**E**) Venn-diagram showing the number of mRNAs that were downregulated by TE in RSK1^KO^ and RSK2^KO^ cells relative to WT cells, and in Torin1-treated WT cells. Two mRNAs that were downregulated in all the comparisons are indicated. (**F**) Summary of the downregulated mRNAs by TE and abundance after Torin1 treatment of LN18^CRISPR^ cells. (**G**) Effect of RSK1 deficiency on mRNAs coding ribosomal proteins relative to WT cells or in the presence of Torin1. (**H**) Effect of RSK2 deficiency on mRNAs coding ribosomal proteins relative to WT cells or in the presence of Torin1. (**I**) WT and RSK1^KO^ cells were treated in the absence or presence of Torin1 as in Figure 3A, and traditional polysome profiles were performed. Total mRNA from the cytosolic extracts and mRNA from fractions of the polysome profiles were extracted and further analyzed by RT-qPCR. (**J**) Distribution of RPLP2 mRNA in the polysome profile. (**K**) Graph showing the fraction of RPLP2 mRNA associated with ≥ 3n polysomes. (**L**) Graph showing total RPLP2 mRNA levels. The mean values of at least four independent experiments (± SEM) are presented.

Changes in the translatome can be mediated by different regulatory modes of gene expression, including translation efficiency (TE; where changes in gene expression are driven by changes in polysome-associated mRNA/ribosome occupancy but not in total mRNA), abundance (which implies congruent changes in total and polysome-associated mRNAs) or buffering (wherein polysome-associated mRNA remains unaltered despite changes in corresponding total mRNA levels) (Supplementary Figure S12A).^20^ Since RSKs phosphorylate proteins associated with translation control, such as eIF4B, their isoform-specific effects on TE were investigated. We observed that the lack of RSK isoforms reduces TE of a significant number of mRNAs (933 and 1220 for RSK1^KO^ and RSK2^KO^ cells, respectively) (Figure 5A, B and Supplementary File S4); the effect on DKO cells was compounded, with 1775 mRNAs showing downregulation in TE (Figure 5C and Supplementary File S4). It was proposed that modulation of TE by RSKs is dependent on mTORC1 activation.^9^ To verify this possibility, we treated LN18^CRISPR^ cells with Torin1. The effect of mTORC1 inhibition on TE was lower than the effect of RSK isoform depletion (Figure 5D and Supplementary File S5). Surprisingly, only a small fraction of the mRNAs with TE reduction in RSK knockout cells were also downregulated after Torin1 treatment (Figure 5E and Supplementary Figure S13A). These data confirm that the effect of RSK on mRNA translation does not involve mTORC1 as a major player. While mTORC1 inhibition showed a reduction in translation of almost a thousand mRNAs in RSK1^KO^ cells, only 16% were affected by Torin1 treatment in RSK2^KO^ cells (Figure 5F, Supplementary Figure S12B, C, and Supplementary File S5). One possibility that can explain this difference is that in RSK2^KO^ cells, mRNAs that are sensitive to Torin1 were already downregulated by TE. This possibility is supported by the fact that several mRNAs coding ribosomal proteins, which are characterized by the presence of a 5′TOP motif,^32^ are already downregulated in RSK2^KO^ cells (Figure 5H) but not in RSK1^KO^ cells (Figure 5G). Like RSK2^KO^ cells, DKO cells also showed a reduced number of transcripts downregulated by TE after Torin1 treatment (Figure 5F, Supplementary Figure S12D, and Supplementary File S5). Additionally, among the most downregulated transcripts in RSK1^KO^ cells, we observed a modest enrichment of transcripts with longer and more structured 5′ UTRs compared to RSK2^KO^ cells (Supplementary Figure S14). This is consistent with RSK1 playing a more prominent role than RSK2 in activating eIF4B, which enhances the processivity of the helicase eIF4A during the translation initiation of transcripts with complex 5′ UTRs.^33^

To validate the results from the translatomic analysis, we performed traditional polysome profiles followed by RT-qPCR from WT and RSK1^KO^ cells in the presence and absence of Torin1 (Figure 5I). In the translatomics, we identified RPLP2 and FIBP as two mRNAs downregulated by TE in RSK1^KO^ and RSK2^KO^ cells, and also after mTORC1 inhibition (Figure 5E). RPLP2 mRNA distribution in the continuous polysome profiles showed a reduced association with polysomes in RSK1^KO^ cells (Figure 5J, K) but its total mRNA levels were maintained the same (Figure 5L). For the FIBP mRNA, although total mRNA levels changed upon Torin1 treatment (Supplementary Figure S15A), this mRNA also showed reduced association with polysomes in RSK1^KO^ cells (Supplementary Figure S15B, C). Furthermore, we also observed a shift in the distribution toward less association with polysomes for SEPHS1 and AP4M1, and to a lesser extent TIMELESS and CDC45 mRNAs, which were classified as downregulated by TE in RSK1^KO^ but not RSK2^KO^ cells (Supplementary Figure S16). YWHAZ mRNA is usually used as a housekeeping reference for RT-qPCR,^34^ and we observed no change in both total mRNA levels (Supplementary Figure S15D) and TE efficiency between WT and RSK1^KO^ cells; however, Torin1 marginally reduced translation (Supplementary Figure S15E, F). Other housekeeping genes, such as GAPDH and β-actin, show important changes in TE under Torin1 treatment (Supplementary Figure S15G-L).

### Identification of a RSK1-specific Translatome Resistant to Torin1

**Figure 6.**
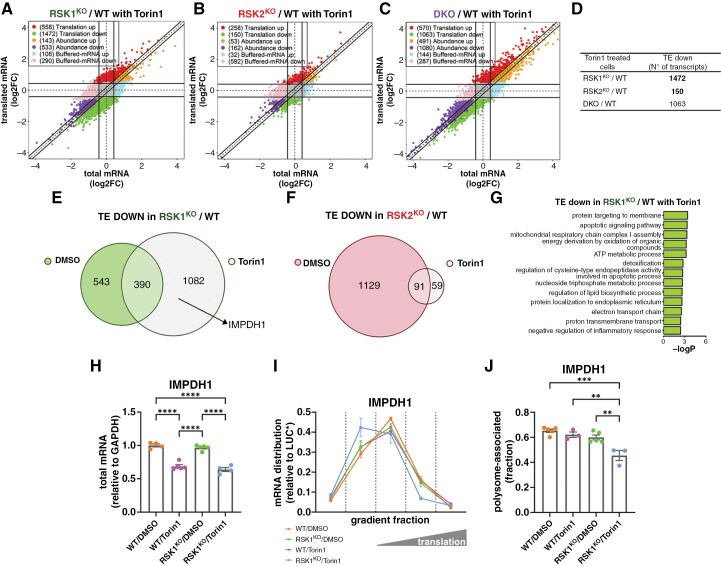
**Torin1-resistant translation is mediated by RSK1 in LN18** ^**CRISPR**^ **cells.** (**A-C**) Graphs showing changes in gene expression and the assigned regulatory modes according to the anota2seq method for the contrasts (**A**) RSK1^KO^/WT, (**B**) RSK2^KO^/WT, and (**C**) DKO/WT in the presence of Torin1. The number of genes classified in each of the modes is indicated within parentheses. (**D**) Summary of the downregulated mRNAs by TE in the presence of Torin1. (**E**) Venn diagram showing the number of mRNAs downregulated by TE in RSK1^KO^ cells relative to WT cells in the absence and presence of Torin1. IMPDH1 mRNA that was tested by RT-qPCR is indicated. (**F**) Venn diagram showing the number of mRNAs downregulated by TE in RSK2^KO^ cells relative to WT cells in the absence and presence of Torin1. (**G**) Graph showing the biological processes associated with mRNAs that were downregulated by TE in RSK1^KO^ cells only in the presence of Torin1 (Torin1-resistant mRNAs). (**H**) Graphs showing total IMPDH1 mRNA levels. (**I**) Distribution of IMPDH1 mRNA in the polysome profile of Figure 5I. (**J**) Graph showing the fraction of IMPDH1 mRNA associated with ≥ 3n polysomes. The mean values of at least four independent experiments (± SEM) are presented. **P < .005 (one-way ANOVA followed by Tukey’s post hoc test).

A striking result was that the effect of Torin1 on TE in RSK1^KO^ cells is more prominent than in both WT and RSK2^KO^ cells (Figure 5D, F and Supplementary Figure S12B, C). Notably, in the presence of Torin1, we noticed a significant reduction in TE for many transcripts in the absence of RSK1 (Figure 6A and Supplementary File S6) but not in the absence of RSK2 (Figure 6B and Supplementary File S6). Interestingly, DKO cells exhibited a similar behavior to RSK1^KO^ cells in the presence of Torin1 (Figure 6C, D and Supplementary File S6). In the absence of Torin1, 933 mRNAs were downregulated by TE in RSK1^KO^ cells; however, this number increased to 1472 in the presence of Torin1 (Figure 6E). In addition, approximately 74% of the mRNAs downregulated by TE in the presence of Torin1 in RSK1^KO^ cells were not affected by the loss of RSK1 in the absence of Torin1. In contrast, while 1220 mRNAs were downregulated by TE in RSK2^KO^ cells in the absence of Torin1, only 150 mRNAs were downregulated in the presence of Torin1 in these cells (Figure 6F). DKO cells show a large downregulation without Torin1, and Torin1 treatment further induced an important downregulation of the TE of another subset of transcripts, although this effect was less evident than in RSK1^KO^ cells (Supplementary Figure S13B). This suggests that mTORC1 inhibition reveals a RSK1-dependent translation control. Remarkably, the Torin1-resistant and RSK1-dependent mRNAs are primarily associated with mitochondrial respiratory processes (Figure 6G and Supplementary File S7). Interestingly, RSK1^KO^ and DKO LN229^CRISPR^ cells exhibited increased cytotoxicity, as measured by the Alamar Blue assay, upon treatment with Torin1 (Supplementary Figure S17).

We analyzed in a traditional polysome profile experiment the distribution of IMPDH1 mRNA, whose TE was reduced in RSK1^KO^ cells only in the presence of Torin1 (Figure 5I). Total mRNA levels of IMPDH1 were reduced to the same extent after Torin1 treatment in WT and RSK1^KO^ cells (Figure 6H); however, in the presence of Torin1, we observed a shift of IMPDH1 mRNA toward the top of the gradient in RSK1^KO^ compared to WT cells (Figure 6I). Unexpectedly, some mRNAs that were not classified as translationally regulated in the presence of Torin1, including SEPHS1, AP4M1, TIMELESS and CDC45 mRNAs, showed a marked re-distribution to the top of the gradient (ie, reduced TE) in Torin1-treated RSK1^KO^ cells compared to Torin1-treated WT cells (Supplementary Figure S18). These findings suggest that the set of mRNAs whose translation depends on RSK1 and is resistant to Torin1 may be broader than what was initially revealed by the genome-wide translatome analysis.

## Discussion

This study investigated the isoform-specific roles of RSK1 and RSK2 in GBM cells, revealing that RSK1 primarily drives phosphorylation of eIF4B and uniquely sustains translation of specific mRNAs involved in cell cycle and DNA repair. These findings particularly uncover a distinct, mTORC1-independent translational program regulated by RSK1, highlighting its potential impact on GBM progression and therapeutic targeting.

An important finding of our article is that the expression of RSK isoforms in GBM cell lines was similar to the patterns of expression in GBM tissues obtained from patients.^10^ One open question from our previous study on RSK isoform expression in GBM patients is whether immune infiltrate cells can also contribute to aberrant high levels of RSK1. While our results strongly support the existence of tumor cells that can indeed contribute to the high RSK1 levels observed in some patients, we cannot yet exclude any contribution from cells of the tumor microenvironment. In fact, it was recently shown that RSK1 is enriched in regions of the tumor expressing microglia signatures.^35^

Previous work of our group indicated that the two most commonly used RSK inhibitors (BI-D1870 and SL0101) showed non-specific effects on the mTORC1 signaling pathway, which contributed to the difficulty in the identification and definition of RSK functions.^16^ The CRISPR/Cas9 technology allowed us to generate RSK isoform-specific knockout cells, which provided highly reliable models to investigate functions previously ascribed to RSKs. We confirmed here that mTORC1 downstream targets are not affected by the loss of RSKs in GBM cells, which challenges the relevance of RSKs as direct activators of mTORC1 (Supplementary Figure S1B). Since PI3K/AKT/mTORC1 signaling is not over-activated in LN229 cells compared to LN18 cells,^16^ we could exclude any masking that mTORC1 over-activation could introduce to RSK effects on mTORC1 in LN18 cells.

Here we also analyzed the RSK-isoform-specific effects on known RSK and mTORC1/S6K shared substrates that control protein synthesis in order to better understand the cross-talk between these two pathways. We previously observed that phosphorylation at S235/236 of RPS6 was mainly dependent on mTORC1 in LN18 and LN229 cells, and to a minor extent on RSKs.^16^ Accordingly, we noticed about 50% reduction of RPS6 phosphorylation only in DKO LN18^CRISPR^ cells but not in the individual knockouts. However, we detected an unexpected increase in RPS6 phosphorylation after PMA treatment in RSK2^KO^ LN229^CRISPR^ cells (Figure 2J, M), which could be associated with the lack of activation of a negative feedback loop.^36^ We previously showed that eIF4B phosphorylation at S422 was resistant to rapamycin in LN18 cells.^16^ Here we observed that eIF4B phosphorylation was resistant to Torin1 treatment in LN229 cells. On the other hand, eIF4B phosphorylation was abolished in DKO cells, indicating that phosphorylation of eIF4B is mainly controlled by RSK1 and not mTORC1 in GBM cells. Importantly, LN229 cells show comparable RSK1 and RSK2 levels, suggesting that eIF4B phosphorylation preference on RSK1 is not due to higher expression of RSK1 than RSK2. Unexpectedly, eEF2K phosphorylation at T366 was not affected by either RSKs or mTORC1 in PMA-treated LN229 cells, implying that a different kinase is responsible for eEF2K phosphorylation at T366. Altogether, our data indicate that any cross-talk between RSK and mTORC1 pathways is rather occurring downstream than upstream of mTORC1 in GBM cells. It is important to note that RSKs can phosphorylate and regulate other factors that control protein synthesis besides those analyzed here, such as PDCD4.^37,38^

Based on the published conclusion that mTORC1 is regulated by RSKs,^9^ it was expected that RSK and mTORC1 effects on translation control would overlap. However, we demonstrated here that at least in GBM cells, mTORC1 activity is not affected by RSKs, and accordingly, we observed little overlap between the set of mRNAs with reduced TE in the absence of RSK isoforms and the set downregulated by mTORC1 inhibition. One of the major mRNA families that are regulated at the level of TE by mTORC1 comprises mRNAs containing 5′TOP motifs, which include the majority of mRNAs coding ribosomal proteins (RPs).^32^ Surprisingly, TE of a substantial number of RP mRNAs showed a reduction in RSK2^KO^ cells, even in the absence of Torin1. On the other hand, RSK1 effect on TE of RP mRNAs was less evident. It has been shown that the protein LARP1 is a direct target of mTORC1 and mediates mTORC1-dependent regulation of 5′TOP-containing mRNAs.^39,40^ Interestingly, S847 and S1056 of LARP1 are located within a consensus phosphorylation site for RSKs^38^ and they can be phosphorylated by S6K1.^41^ It will be important to verify whether RSKs can also phosphorylate these sites in LARP1 to modulate 5′TOP mRNA translation.

mTORC1 is necessary to regulate mitochondrial function and biogenesis through the translation of mitochondrial-related mRNAs.^42,43^ However, RSK2 deficiency, but not RSK1, also resulted in reduced translation of mitochondrial-related mRNAs, even when mTORC1 is active. Unexpectedly, our data revealed that RSK1 can maintain translation of mRNAs in the absence of mTORC1 activation, and those RSK1-dependent mTORC1-resistant mRNAs show an enrichment in mRNAs related to mitochondrial respiration. This implies that other factors besides mTORC1 could regulate mitochondrial-related mRNA translation, which is further supported by the role of eIF4G1 as a regulator of mitochondrial oxidative phosphorylation through the translation of mitochondrial-related mRNAs.^44^ Our data also suggest an isoform switch from RSK2 to RSK1 for the control of mitochondrial-related mRNAs, which depends on the levels of activity of mTORC1. It will be important to investigate if the mTORC1-resistant RSK1-dependent translation mechanism has a role in the observed resistance of GBM to mTORC1 inhibition.^45^

In this article, we show that eIF4B phosphorylation is preferentially mediated by RSK1, rather than by RSK2 or through mTORC1. This may explain, at least in part, some of the differences observed in the translatomes of RSK1^KO^ and RSK2^KO^ cells. Accordingly, we found a preferential, albeit modest, downregulation of transcripts with complex 5′UTRs in RSK1^KO^ cells. This suggests that transcripts requiring higher eIF4A processivity, promoted by eIF4B, are specifically targeted by RSK1. Interestingly, AURKB, CDC25C, AP4M1, and SHMT1, some of the most strongly downregulated transcripts by translation efficiency (TE) in RSK1^KO^ cells, harbor eIF4B-binding motifs in their 5′UTRs.^46^

A recent article by Yang et al. analyzed the transcriptome of RSK1^KO^ and RSK2^KO^ U251MG GBM cells but not DKO cells.^30^ The article showed that RSK1 depletion in U251MG cells was associated with reduced expression of genes related to cell cycle regulation, DNA replication, and repair, which is consistent with our results presented here with the LN18 cells (Supplementary File S8). Notably, the article also characterized CDC45 as one of the RSK1-regulated genes. Contrary to RSK1 depletion, the effects of RSK2 depletion on gene expression in U251MG cells were different from those observed in RSK2^KO^ LN18^CRISPR^ cells (Supplementary File S8). It is important to note that in our study, the gene-expression changes observed in RSK2^KO^ cells were essentially restricted to the translatome and supported by changes in TE but not in the transcriptome. In this manner, the analysis of the translatome provided a more in-depth readout on the effects of RSK2 on gene expression and uncovered effects of RSK2 on mitochondria-related gene expression not observed in the transcriptome of RSK2^KO^ U251MG cells.

Several substrates of RSKs have been reported to modulate gene expression programs, particularly at the transcriptional level. These include transcription factors such as CREB and SRF.^12^ Furthermore, both RSK1 and RSK2 have been shown to form regulatory complexes with CREB-binding protein (CBP), which, through its histone acetyltransferase activity, mediates transcriptional coactivation.^47,48^ Another factor that may contribute to the cell cycle-related regulation of gene expression is the fact that key cell cycle regulators, such as CDKN1B and CDC25 isoforms, are also direct substrates of RSKs.^49–51^ A key challenge will be to define the isoform-specific substrate and binding partners preferences of RSKs, to clarify how each isoform contributes to the regulation of gene expression programs.

The determinants of the distinct functions of RSK1 and RSK2 remain poorly understood. One possibility is that different binding partners mediate the unique functions of each isoform. For instance, RSK1 has been shown to interact with PKA subunits, which influence its subcellular localization and activation. Notably, depletion of the regulatory subunit PKARIα increased the activation of RSK1 but did not affect RSK2 or RSK3.^52^ Conversely, the protein PEA-15 interacts with and regulates the subcellular localization and activity of RSK2 but not RSK1.^53^ While our study demonstrates that distinct gene expression programs are mediated by RSK1 and RSK2, further research is needed to uncover the specific molecular mechanisms underlying these differences.

In conclusion, we provide mechanistic and translatomic data on the isoform-specific functions of RSKs in GBM, contributing to a better understanding of their role in brain cancer.

## Supplementary Material

vdaf144_suppl_Supplementary_Figures_S1-S18_Tables_S2_Files_S1-S8

## Data Availability

The Clariom^TM^ D human microarray data from this study were deposited in the NCBI Database of GEO with accession number GSE263893.
