# Supplementary material for: RSK1 and RSK2 modulate the translatome of glioblastoma cells in an isoform-specific and mTORC1 independent manner
Source: Neurooncol Adv. 2025 Jul 11;7(1):vdaf144. doi: 10.1093/noajnl/vdaf144 (PMC12536492; doi:10.1093/noajnl/vdaf144)
Supplement: vdaf144_suppl_Supplementary_Figures_S1-S18_Tables_S2_Files_S1-S8 [file vdaf144_suppl_supplementary_figures_s1-s18_tables_s2_files_s1-s8.zip › Supplementary Material.pdf]

Supplementary Material for

RSK1 and RSK2 modulate the translatome of glioblastoma cells in an isoform-specific and mTORC1 independent manner

Martín Roffé, Danielle P. Nascimento, Paula B. Nunes, Luana C. Soares, Arielly D. H. Alves, Ali Hamraghani, Yeganeh Almasi, Zakia Djaoud, Glaucia N. M. Hajj, Vilma R. Martins, Nahum Sonenberg, Tommy Alain

This PDF file includes:

Supplementary Materials and Methods

Supplementary Tables S1 to S3

Supplementary Figures S1 to S19

Supplementary Legends for Files S1 to S8

Supplementary References

SUPPLEMENTARY MATERIAL AND METHODS

Cell culture

LN18 (ATCC® CRL-2610™), LN229 (ATCC® CRL-2611™), U87MG (ATCC® HTB-14™), A172 (ATCC® CRL-1620™) and U118MG (ATCC® HTB-15™) cells were cultured in Dulbecco's Modified Eagle Medium (DMEM), high glucose, GlutaMAX, pyruvate (Gibco®), supplemented with 10% fetal calf serum. LN18 cells were transfected to express influenza hemagglutinin (HA)-tagged RSK3 (HA-RSK3) or RSK4 (HA-RSK4) using Lipofectamine® 2000 (Invitrogen™). These plasmids were described previously.[1]

Western blot

Cells were lysed in a lysis buffer composed of 50 mM Tris–HCl (pH 8.0), 150 mM NaCl, 1% Nonidet P-40, 0.5% sodium deoxycholate, 1 mM EDTA and 1 mM EGTA, and supplemented with a protease and phosphatase inhibitor cocktail (Roche and Thermo Fisher Scientific, respectively). Immunoblots were performed as described previously.[2] The primary antibodies used are listed in Supplementary Table S1.

Generation of RSK-knockout GBM cells using CRISPR/Cas9

The CRISPR/Cas9 system was employed to edit the genome of LN18 and LN229 cells following the protocol published by Ran et al.[3] with minor modifications. Briefly, sgRNA guides targeting *RSK1* (*RPS6AK1*) and *RSK2* (*RPS6KA3*) genes were designed using the guide design tool at http://crispr.mit.edu/. The DNA oligonucleotides encoding the sgRNAs (see Supplementary Table S2) were cloned into the pSpCas9(BB)-2A-Puro (PX459) V2.0 plasmid (Addgene), which encodes the wild-type spCas9 and a puromycin resistance cassette. LN18 or LN229 cells were transfected with the plasmid using Lipofectamine® 2000 in Opti-MEM medium following the manufacturer's protocol. Two days after transfection, cells containing the plasmid were selected with puromycin. Following selection, individual clones were expanded, and the expression of RSK1 and RSK2 was assessed by Western blotting. To generate double knockout (DKO) cells, cells were transfected with two plasmids for sgRNA

targeting either RSK1 (sgRSK1) or RSK2 (sgRSK2). Clones of cells transfected with an empty plasmid lacking sgRNAs were used as controls (WT).

Immunoprecipitation

LN18 cells were lysed in the same lysis buffer for Western blotting, supplemented with 1X protease inhibitor cocktail (cOmplete^{TM}, Roche). Extracts were pre-cleared with Protein A-agarose beads (Roche), and then divided into three fractions for incubation with different antibodies. Cleared extracts were incubated with anti-RSK1 (sc-231, Santa Cruz) or anti-RSK2 (NBP1-85552, Novus Biologicals) antibodies pre-bound to Protein A-agarose, in washing buffer (50 mM Tris-HCl pH 8.0, 150 mM NaCl, 0.5% Triton X-100), overnight at 4°C. A fraction of the extracts was incubated only with Protein A-agarose to control for non-specific binding. Beads were washed 5X with washing buffer and eluted with 2X Laemmli buffer (Sigma). A fraction (10%) of the eluate was reserved for Western blotting to estimate immunoprecipitation (IP) efficiency and the remaining eluate was used for SDS-PAGE, followed by silver staining (Pierce).

Non-linear Polysome Profiling

Prior to cell harvest, 100 μg/ml cycloheximide (Sigma Aldrich) was added to the culture medium and incubated for 7 minutes at 37°C. Cells were then washed twice in PBS containing the same concentration of cycloheximide, scraped, and collected in a microcentrifuge tube. The cells were centrifuged at 500 x*g* for 3 minutes and 4°C. Subsequently, cells were lysed in lysis buffer containing 20 mM Tris-HCl pH 7.5, 100 mM NaCl, 10 mM $MgCl_2$, 1% Triton X-100 (Sigma Aldrich), 1 mM DTT, 100 μg/ml cycloheximide, 40 U/ml RNasin (Promega) and EDTA-free protease inhibitor cocktail (Thermo). The lysates were cleared by centrifugation at 10,000 x*g* for 10 minutes at 4°C. A fraction of the supernatant was used for total mRNA extraction, and the remaining extract was applied to a sucrose non-linear gradient made in 20 mM Tris-HCl pH 7.5, 100 mM NaCl, 5 mM $MgCl_2$, and 1 mM DTT, as previously described [4]. The gradients were centrifuged at 35,000 RPM for 2 hours at 4°C in a SW41Ti rotor and Optima XE-90 Ultracentrifuge (Beckman Coulter). Absorbance at 254 nm was read from the top of the gradient using a continuous UV detector (BioLogic LP, Bio-Rad), while fractions were collected.

Efficiently translated mRNAs (> 3n polysomes) were concentrated and isolated from one or two fractions of the gradient (Fig. 3C). Translation levels of LN18^{CRISPR} cells were estimated by the ratio of the area under the peak of efficiently translated mRNAs to the area under the peaks of poorly translated mRNAs and monosomes (Fig. 3C, D).

Microarray Assay

RNA was purified from the fractions of the non-linear gradient containing polysome-associated mRNA and from an aliquot of the original cytosolic extracts (total mRNA) using TRI-Reagent® (Sigma Aldrich) and the Direct-zol™ RNA MiniPrep kit (Zymo Research). The integrity of the RNA was evaluated using the RNA 6000 Pico Chips kit in a 2100 Bioanalyzer (Agilent Technologies). Both total and polysome-associated mRNA were subjected to the Clariom™ D human microarray (Thermo Fisher Scientific) according to the manufacturer's instructions.

Microarray data processing

The CEL files were normalized and summarized at the gene level using SST-RMA algorithm from the Transcriptome Analysis Console software (Thermo). Normalized expression and DABG (detection above background) values were retrieved from the CHP files using the *affxparser* package. Transcript clusters with DABG ≥ 0.05 in at least one of the samples were excluded. Data were annotated using the *clariomdhumantranscriptcluster.db* package in R. Only coding transcript clusters with a corresponding RefSeq ID for protein ('NP') were retained, and transcript clusters for the same gene were collapsed (based on max intensity) using the WGCNA package.[5] One sample of the biological triplicates of the DMSO-treated polysome-associated RNA of WT LN18^{CRIPSPR} cells required further processing. Since no significant changes were observed in overall translation levels of DMSO treated WT, RSK1 and RSK2 cells in comparison to DKO and Torin1-treated cells, we re-scaled the problematic sample relative to DMSO-treated WT, RSK1^{KO}, and RSK2^{KO} cells. First, the medians at the transcript and cell type level were obtained, and the median absolute deviation (MAD) for each transcript were calculated. Second, the transcripts from the problematic sample were separated into three sets: transcripts >= 0.75*MAD; transcripts <= 0.75*MAD; and transcripts between those limits. The three sets were re-scaled separately

using the *normalizeBetweenArrays* (method = scale) function of the limma package. Finally, the transcripts from the different sets were combined and re-scaled using *normalizeBetweenArrays* (method = quantile) function.

Gene expression analysis

Normalized polysome-associated and total mRNA were analyzed by the anota2seq package.[6] Anota2seqSelSigGenes filter parameters were set as follows: maxSlopeTranslation = 2; minSlopeTranslation = −1; maxSlopeBuffering = 1; minSlopeBuffering = −2; deltaPT = log2(1.2); deltaTP = log2(1.2); deltaP = log2(1.5); deltaT = log2(1.5), maxP = 0.05.

Linear polysome profiling

LN18^{CRISPR} cells grown on two 150-mm dishes were serum-starved for 48 hours and then pre-treated with 250 nM Torin1 (or DMSO) for 30 minutes before stimulation with 10% serum for 6 hours. Cells were incubated with 100 μg/ml cycloheximide for 7 minutes at 37°C, washed with PBS containing 100 μg/ml cycloheximide, scraped, and sedimented by centrifugation. Cytosolic extract preparation was performed as previously described with minor modifications.[7] Briefly, cells were incubated in 324 μl hypotonic buffer (5.55 mM Tris-HCl pH 7.5; 2.78 mM MgCl2; 1.67 mM KCl; 1.11 mM DTT; 111 μg/ml cycloheximide; 111 U/ml RNasin (Promega); 0.11X protease inhibitor cocktail (EDTA-Free; Roche)) for 5 minutes with 10 seconds vortex. 18 μl of 10% Triton X-100 and 18 μl of 10% Sodium Deoxycholate were added, followed by another 5 minutes of incubation including 10 seconds vortex. Extracts were clarified by centrifugation for 10 min at 10,000 x*g* in a refrigerated microcentrifuge. A fraction of the supernatant was stored for total RNA analysis, and the remaining supernatant was applied to 10–50% sucrose gradients, prepared in 20 mM Tris-HCl pH 7.5, 100 mM KCl, 5 mM $MgCl_2$, 1 mM DTT. Gradients were centrifuged in a SW41 Ti rotor (Beckman) at 36,000 rpm for 90 minutes and the absorbance was measured in a continuous flow while collecting 750 μl fractions.

RT-qPCR

The 15 fractions of the gradient were pooled into 5 fractions from the top to the bottom of the gradient. For that, 250 µl from 3 fractions were combined to a final volume of 750 µl. Luciferase (LUC) mRNA (Promega) was added to each of the pooled fractions. The pooled fractions were extracted following the TRIzol protocol using 1 ml TRIzol (Invitrogen) and 200 µl chloroform. 1 µl GlycoBlue™ Coprecipitant (Thermo) was added before precipitation of RNA with isopropanol. The same volume of RNA was employed for retrotranscription with the iScript Advanced cDNA Synthesis kit (Bio-Rad), and qPCR was performed using the SsoAdvanced Universal SYBR Green Supermix (Bio-Rad). The addition of luciferase (LUC) mRNA was used to correct differences during the process of RNA extraction from the pooled fractions and RT-qPCR. It is important to note that there exists an inherent error in both the addition of LUC mRNA and the downstream steps, which can complicate the visualization of differences in the determination of translation rates (Supplementary Fig. S19). Thus, we performed a correction to minimize it. We calculated the LUC/ACTB mRNA ratio in every pooled fraction. Since in theory the LUC mRNA levels should be equal in each of the fractions and replicates, we calculated the deviations observed in the replicates and then we corrected the LUC mRNA values (LUC*) for the other mRNAs. It is important to mention that besides ACTB, we tested several mRNAs and their combinations and obtained similar results. For ACTB, we used LUC/RPLP2 ratio to obtain LUC*.

Total RNA was extracted from the cytosolic lysate with the RNeasy® Mini Kit (Qiagen) and used for RT-qPCR as mentioned above. For the analysis of RSK isoform expression in GBM cell lines, total RNA was extracted by TRIzol and Direct-zol™ RNA MiniPrep kit (Zymo Research). RT-qPCR was performed using the GoTaq® Probe 2-Step RT-qPCR System (Promega). The sequences of qPCR primers are given in Supplementary Table S3.

Cell proliferation and viability

Cell proliferation was monitored by measuring cell confluence using the Incucyte Live-Cell Imaging System (Sartorius) and analyzed using Incucyte software. DNA synthesis was assessed via bromodeoxyuridine (BrdU) incorporation using the BrdU Cell Proliferation Assay Kit (Cell Signaling Technology), according to the manufacturer's instructions. Cell viability was determined using the

Alamar Blue method. Briefly, cells were incubated with 14 μg/ml Resazurin in culture medium and fluorescence was measured at 530 nm excitation and 590 nm emission using a microplate reader.

Statistical analysis and Graphics

One-way ANOVA followed by Tukey's post hoc test or Student's t-test were applied after assuming normality. $P < 0.05$ were considered statistically significant (* or $^{\#}$). Pearson's and Spearman's coefficients for correlations are indicated in the graphs alongside with the *P-values* in parentheses. Statistical analysis and graphs were performed using GraphPad Prism or RSTUDIO with R version 4.2.1.

Analysis of Yang *et al.* dataset

CEL files from Clariom S Human Array (Affymetrix) associated with the study from Yang *et al.*[8] were obtained from the Gene Expression Omnibus (GEO) repository, accession number GSE213592, and processed in the same manner as the microarrays of this study.

SUPPLEMENTARY TABLES

Table S1. List of primary antibodies used for western blot.

Antibody	Source	Catalog Number
P(S380)-RSK1	Cell Signaling	#9341
RSK1	Santa Cruz	sc-393147
RSK2	Santa Cruz	sc-9986
RSK3	Santa Cruz	sc-1431
RSK3	Cell Signaling	#9343
RSK4	Santa Cruz	sc-100424
β-actin	Sigma	A5441
PTEN	Cell Signaling	#9188
α-tubulin	Sigma	T9026
P(T202/Y204)-ERK1/2	Cell Signaling	#9101
ERK1/2	Cell Signaling	#9102
P(S1798)-TSC2	Santa Cruz	sc-293149
TSC2	Santa Cruz	sc-271314
P(T389)-S6K1	Cell Signaling	#9234
S6K1	Cell Signaling	#2708
P(S235/236)-RPS6	Cell Signaling	#4856
RPS6	Santa Cruz	sc-74459
P(S422)-eIF4B	Cell Signaling	#3591
eIF4B	Santa Cruz	sc-390912
P(T37/46)-4E-BP1	Cell Signaling	#9459
4E-BP1	Cell Signaling	#9644
P(S366)-eEF2K	Cell Signaling	#3691
eEF2K	Cell Signaling	#3692
PLK1	Cell Signaling	#4513
GAPDH	abcam	ab8245

Table S2. sgRNA oligonucleotide inserts cloned into the pSpCas9(BB)-2A-Puro (PX459) V2.0 vector.

Gene	Oligo	Sequence (5′-3′)	Score*
RSK1	top	CACCGgcactagctccatgagcggcc	80
	bottom	AAACggccgctcatggagctagtgcC	
RSK2	top	CACCGgagcccgtccgacagcgctg	86
	bottom	AAACcagcgctgtcggacgggctcC	

*Score provided by the guide design tool. Scores closer to 100 show less off-target effects.

Table S3. List of primers used for RT-qPCR.

mRNA	Forward primer (5′-3′)	Reverse primer (5′-3′)
RSK1	GCCCTGTTCAAGCGGAATC	GTGGAGTAGAAGACATGCCG
RSK2	TTTCAGACACAGCAAAGGACC	GAGCACAAGAGCAGCAGTCA
RSK3	AAGCGCCATCCCTTCTTTGT	TGCCCACTGCTGGTTTGAA
RSK4	GGGATATGATGCTGCTTGTGA	AAATGGAGTGTAGCCAGCCAA
TBP	TATCCCTCCCCCATGACTCC	TGCGGTACAATCCCAGAACT
ACTB	TCCTTCCTGGGCATGGAGTC	TTCATTGTGCTGGGTGCCA
AP4M1	ACTCTCCGATGACCTCCCCT	CATTGAGGGCTTGGCTCTTTG
CDC45	TCTCCTTTGAGTATGACCTCCG	CTTGAACCTGGCTGCGGTAT
FIBP	GAAAGCTGGGCGTCTTCTCT	GGCAGGGTTCCACAAACTTC
GALK1	ACCGCTCACTCAGAGACGAC	CATGCGGCTGCCATAAACC
GAPDH	AGCCGCATCTTCTTTTGCGT	CCCAATACGACCAAATCCGTTG
IMPDH1	GCTACGTGCCCGAGGATG	GGGAGAATCAGGAAGTCGTTGT
RPLP2	ACGTCATTGCCCAGGGTAT	AGGCCAAATCCCATGTCATCA
SEPHS1	AATGACCGACAGGGAAAGGG	GGGTTGGCAGACAGTGGTAG
SHMT1	GAGTAACCGGCAGAGGGTTG	CTCAGTCCCGCCATAGTATCTCT
TIMELESS	CAGCCGCATCATCAAGAACAAT	CTCCGACAGAATCGCTCCAA
YWHAZ	GACTGGGTCTGGCCCTTAAC	TGGCTTCATCAAAAGCTGTCT
Luciferase (LUC)	GCCAGTCAAGTAACAACCGC	TTGGACTTTCCGCCCTTCTT

SUPPLEMENTARY FIGURES

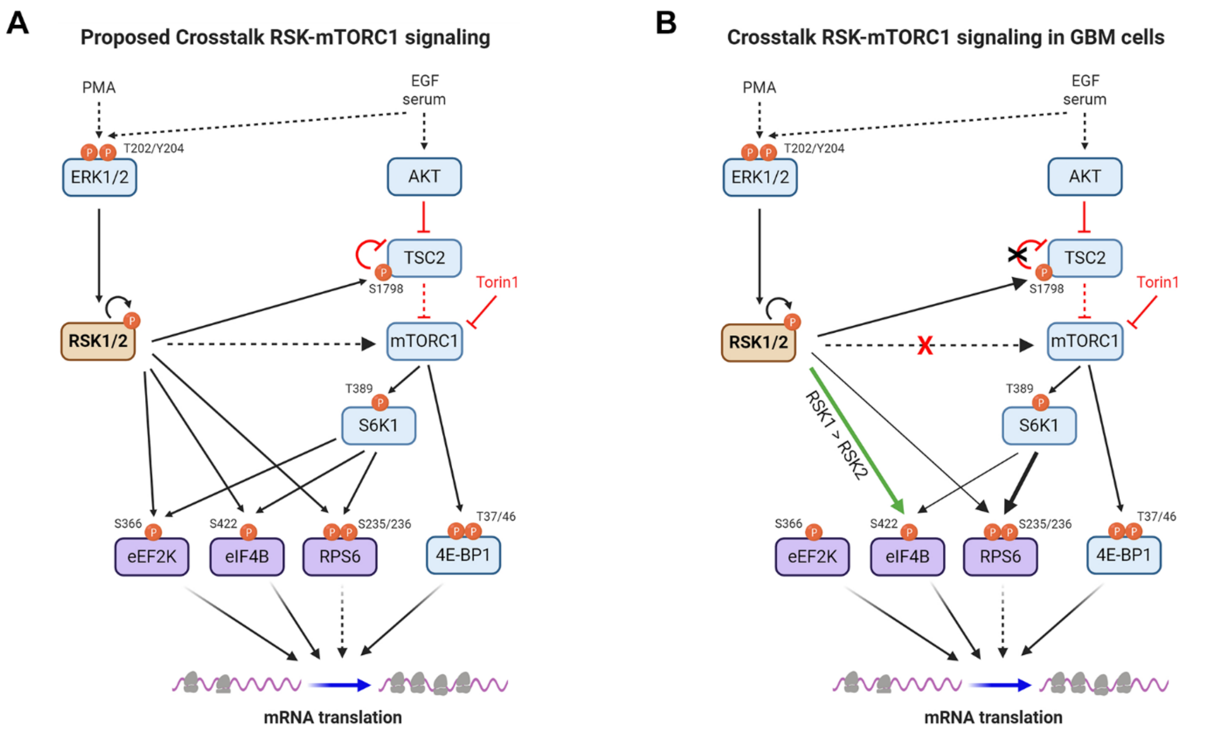

Figure S1. Cross talk RSK-mTORC1. (**A**) Scheme of the proposed cross-talk between ERK/RSK and mTORC1/S6K signaling pathways. ERK1/2 phosphorylation of RSK1 and RSK2 leads to autophosphorylation, which is necessary for RSK activation. It was proposed that RSKs can activate mTORC1 by directly phosphorylating TSC2, inhibiting the tuberous sclerosis complex, which results in mTORC1 activation.[9] Additionally, RSKs could activate mTORC1 by phosphorylating one of its components, Raptor (dashed arrow).[10] Torin1 is an ATP-competitive inhibitor of mTOR.[11] RSKs can also directly phosphorylate substrates that are shared with S6K1, which are related to mRNA translation control, including eIF4B[12], RPS6[13] and eEF2K[14]. Created in BioRender. Roffe, M. (2025) https://BioRender.com/tq1fs4b. (**B**) Scheme of the cross-talk between ERK/RSK in GBM cells lines. Although RSKs can phosphorylate S1798 of TSC2, we did not observe mTORC1 activation by RSKs. RSK1 is primarily responsible for eIF4B phosphorylation in GBM cell lines. RPS6 phosphorylation at S235/236 is mainly mediated by mTORC1 and to a lesser extent by RSKs. We did not observe either RSK- or mTORC1-mediated phosphorylation of eEF2K. Created in BioRender. Roffe, M. (2025) https://BioRender.com/mkzv3wr.

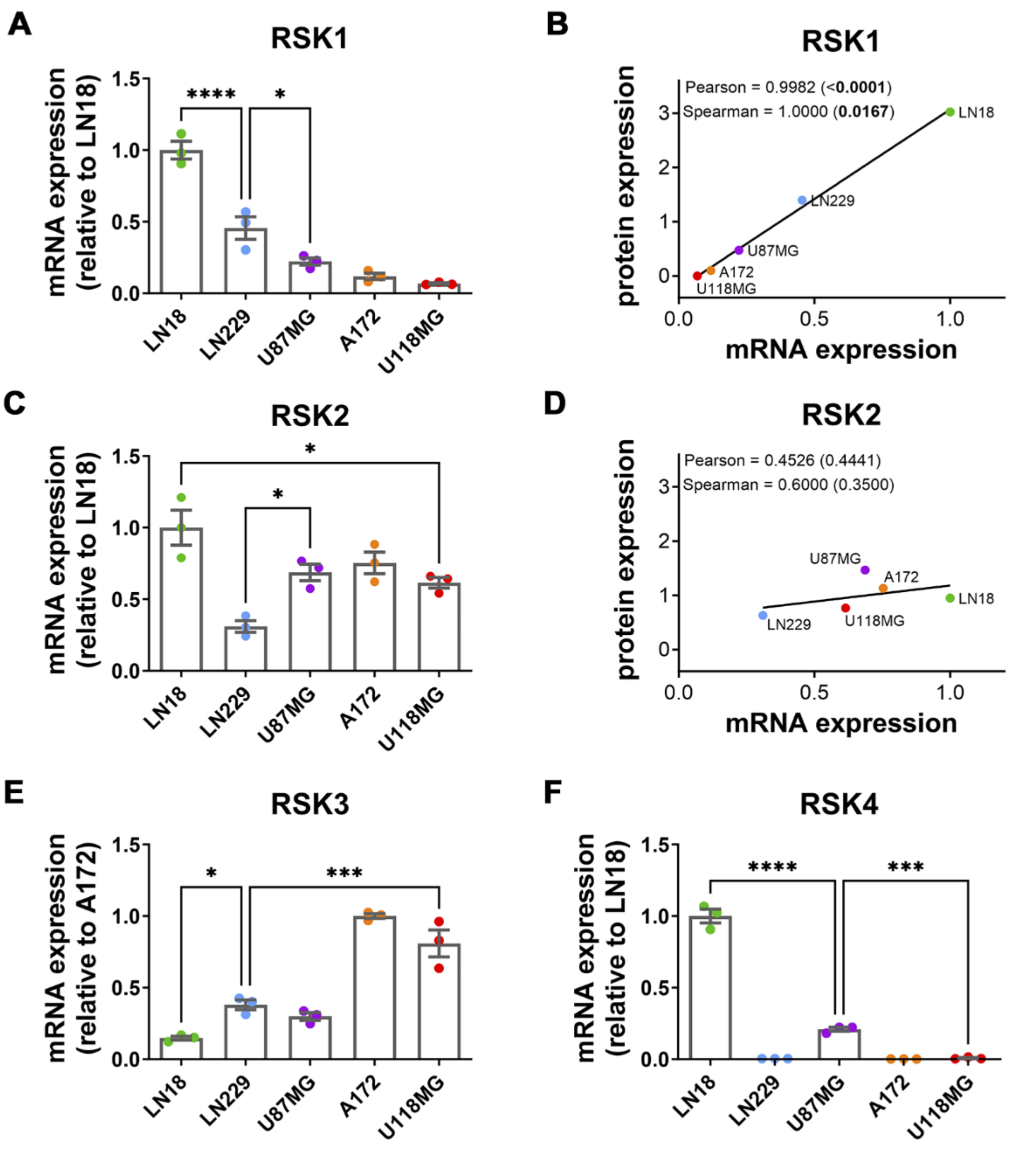

Figure S2. mRNA expression levels of the RSK isoforms in GBM cells. (**A**) RT-qPCR for RSK1 mRNA. Expression was normalized to TBP mRNA. (**B**) Correlation between RSK1 mRNA and protein expression (from Fig. 1C). (**C**) RT-qPCR for RSK2 mRNA. Expression was normalized to TBP mRNA. (**D**) Correlation between RSK2 mRNA and protein expression (from Fig. 1D). (**E**) RT-qPCR for RSK3 mRNA. Expression was normalized to TBP mRNA. (**F**) RT-qPCR for RSK4 mRNA. Expression was normalized to TBP mRNA. Pearson and Spearman correlation coefficients are presented, along with the corresponding *P* values in parentheses. *$P < 0.05$ (one-way ANOVA followed by Tukey's post hoc test). Only selected significant comparisons are showed.

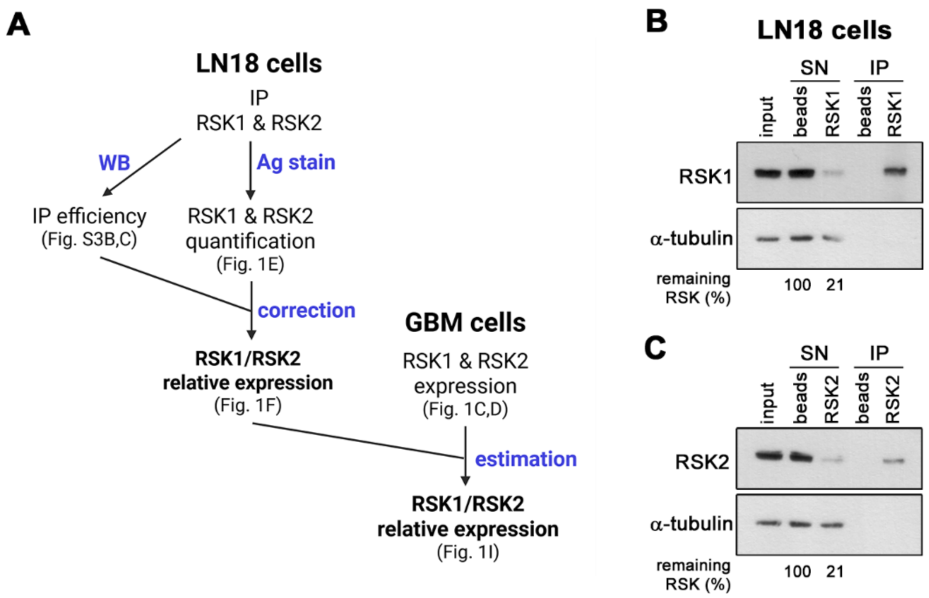

Figure S3. RSK1 and RSK2 stoichiometry in GBM cells. (**A**) Protocol schematic for estimating RSK1 and RSK2 relative content in LN18 cells. RSK1 and RSK2 were immunoprecipitated (IP) from LN18 cell extracts. Western blots were performed to estimate IP efficiency by measuring the non-immunoprecipitated proteins remaining in the supernatants. Immunoprecipitated RSK1 and RSK2 were separated on a polyacrylamide gel and silver (Ag)-stained (Fig. 1E). RSK1 to RSK2 relative expression was estimated by quantifying band intensities in the silver-stained gel, with values corrected for IP efficiency. RSK1/RSK2 relative values obtained for LN18 cells were combined with the results of Fig. 1C, D to estimate relative RSK1/RSK2 content in other GBM cell lines. Created in BioRender. Roffe, M. (2025) https://BioRender.com/3hjezw6. (**B**) Western blot was used to determine RSK1 IP efficiency from LN18 cells, with α-tubulin used as a loading control. A fraction of the immunoprecipitations was also included. (**C**) Western blot used to determine RSK2 IP efficiency from LN18 cells, with α-tubulin used as a loading control. A fraction of the immunoprecipitations was also included.

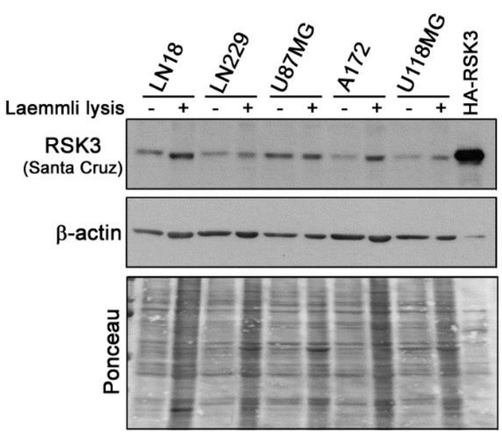

Figure S4. RSK3 expression in GBM cell lines after lysis with Laemmli loading buffer. It has been previously reported that RSK3 can be challenging to extract during cell lysis for western blotting.[15] To rule out this possibility, we lysed the cells directly in Laemmli sample buffer to improve protein extraction. In that manner, cell extracts were prepared either with lysis buffer or Laemmli loading buffer. Equivalent amounts of cell extracts were used for western blot to determine the protein levels of RSK3. A *Santa Cruz* antibody was used, and the western blots were over-exposed. As positive controls, we included extracts obtained from LN18 cells that were transiently transfected to express HA-RSK3. Three times less extract of transfected cells was used. The Ponceau-stained membrane was used as a loading control. Despite using the Santa Cruz antibody, we did not observe an improvement in RSK3 detection, leading us to conclude that RSK3 is expressed at very low levels or not at all in the five cell lines tested.

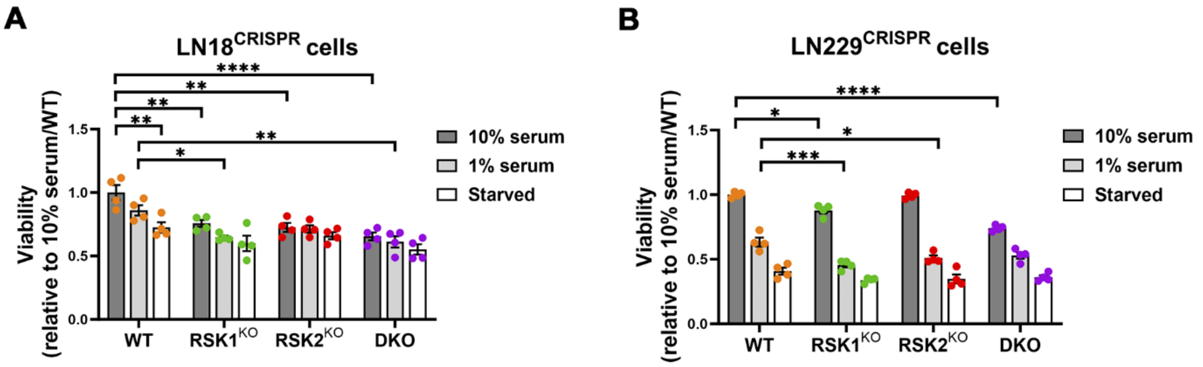

Figure S5. Viability of LN18^{CRISPR} and LN229^{CRISPR} cells. (**A**) Cell viability was measured for LN18^{CRISPR} cells in growing conditions (10% serum) or under serum deficiency. The mean values of four independent experiments (±SEM) are presented. (**B**) Cell viability was measured for LN229^{CRISPR} cells in growing conditions (10% serum) or under serum deficiency. The mean values of three independent experiments (±SEM) are presented.

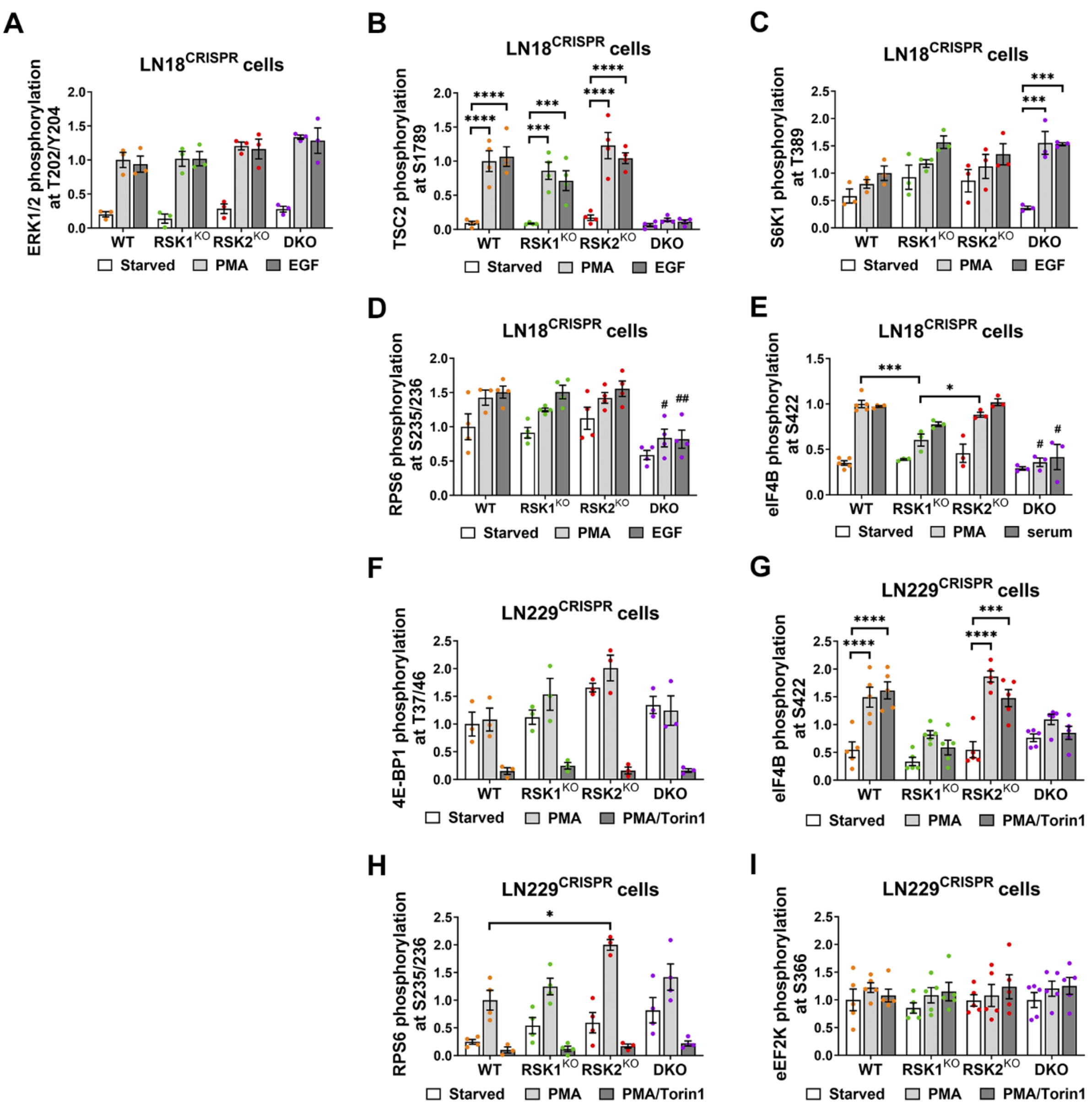

Figure S6. Quantification of phosphorylation levels in western blots of LN18^{CRISPR} and LN229^{CRISPR} cells. (**A-E**) Graph showing the quantification of (**A**) ERK1/2, (**B**) TSC2, (**C**) S6K1, (**D**) RPS6 and (**E**) eIF4B phosphorylation from western blots of LN18^{CRISPR} cells shown in Fig. 2B. The mean values of three independent experiments (±SEM) are presented. (**F-I**) Graph showing the quantification of (**F**) 4E-BP1, (**G**) eIF4B, (**H**) RPS6 and (**I**) eEF2K phosphorylation from western blots of LN229^{CRISPR} cells shown in Fig. 2F. The mean values of three independent experiments (±SEM) are presented.

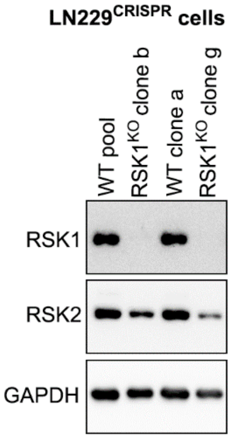

Figure S7. RSK1^{KO} LN229^{CRISPR} cells show a slight reduction of RSK2 expression. RSK1 and RSK2 expression in two different clones of RSK1^{KO} cells were compared to a pool and an individual clone of WT LN229^{CRISPR} cells by western blot. GAPDH was used as a loading control.

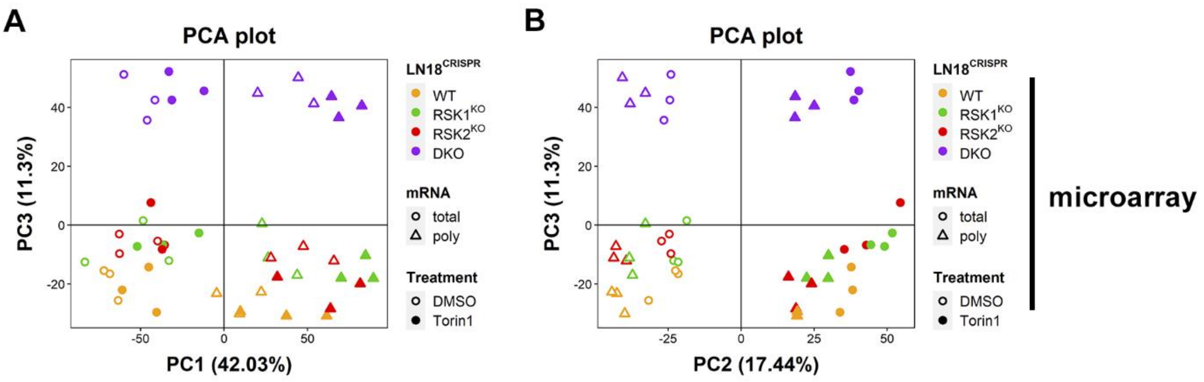

Figure S8. PCA plots for LN18^{CRISPR} cells. Total and polysomes-associated (poly) mRNA obtained from LN18^{CRISPR} cells were analyzed by microarray, and the expression data were used to perform principal component analysis (PCA). (**A**) The plot shows PC1 and PC3. (**B**) The plot shows PC2 and PC3.

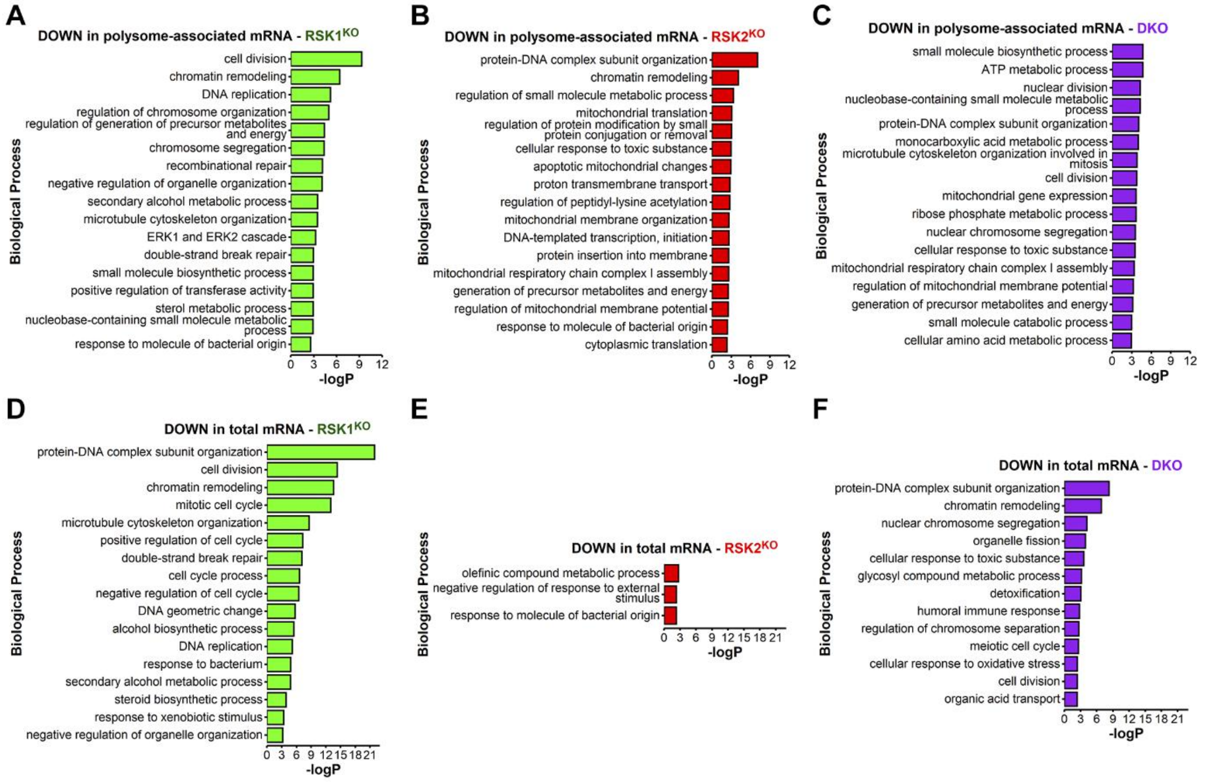

Figure S9. RSK isoforms effects on the transcriptome and translatome. (**A-C**) Downregulated mRNAs in the translatome (polysomes-assocaited mRNA) of knockout LN18^{CRISPR} cells compared to WT LN18^{CRISPR} cells were used to verify the downregulated biological processes for (**A**) RSK1^{KO}, (**B**) RSK2^{KO}, and (**C**) DKO LN18^{CRISPR} cells. (**D-F**) Downregulated mRNAs in the transcriptome (total mRNA) of knockout LN18^{CRISPR} cells compared to WT LN18^{CRISPR} cells were used to verify the downregulated biological processes for (**D**) RSK1^{KO}, (**E**) RSK2^{KO}, and (**F**) DKO LN18^{CRISPR} cells. A cutoff < 0.005 for the hypergeometric test of Gostats package was applied.

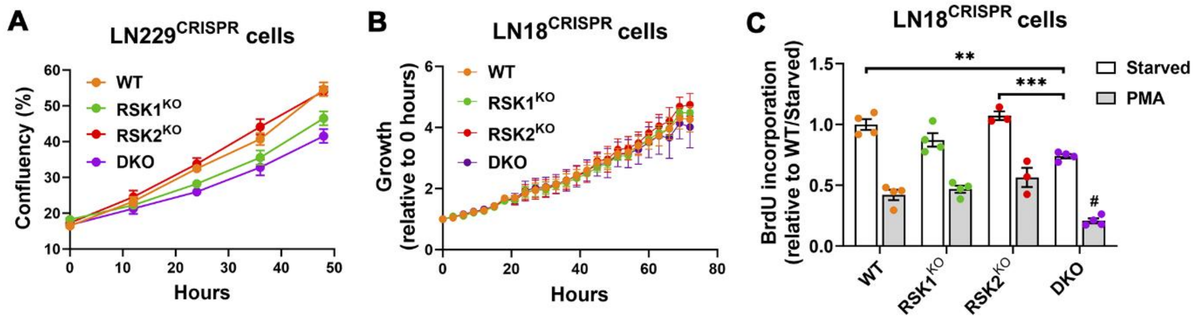

Figure S10. Proliferation of LN18^{CRISPR} and LN229^{CRISPR} cells. (**A**) LN229^{CRISPR} cell growth in medium containing 10% serum was monitored by measuring the confluence using the Incucyte live-cell imaging system. The mean values of four independent experiments (±SEM) are presented. (**B**) LN18^{CRISPR} cell growth in medium containing 10% serum was monitored by measuring the confluence using the Incucyte live-cell imaging system. The mean values of four independent experiments (±SEM) are presented. (**C**) LN18^{CRISPR} cells were serum-starved for 48 hours and then left for another 16 hours in starvation medium or with PMA. BrdU incorporation to the DNA was measured. The mean values of at least three independent experiments (±SEM) are presented.

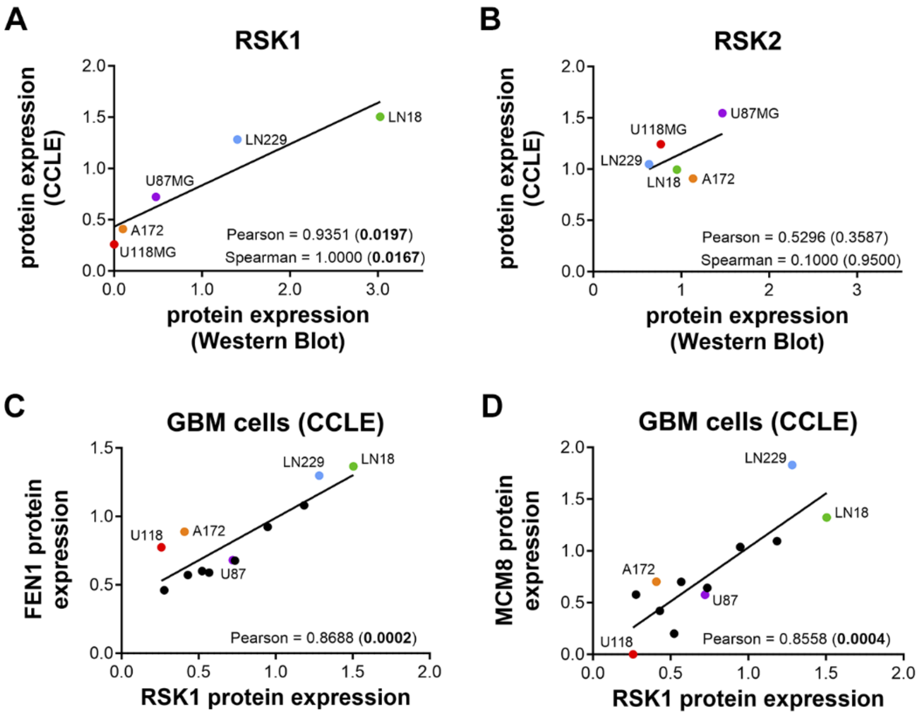

Figure S11. Correlation of RSK1 expression with proteins encoded by mRNAs downregulated in the translatome of RSK1-deficient cells. (**A**) Correlation between RSK1 protein levels analyzed by western blot (Fig. 1C) and RSK1 expression from quantitative proteomics data of the CCLE.[16] Pearson and Spearman correlation coefficients are presented, along with the corresponding *P* values in parentheses. (**B**) Correlation between RSK2 protein analyzed by western blot (Fig. 1D) and RSK2 expression from quantitative proteomics data of the CCLE. Pearson and Spearman correlation coefficients are presented, along with the *P* values in parentheses. (**C**) Correlation between RSK1 and FEN1 protein expression in 12 GBM cells lines from the CCLE. Pearson correlation coefficient is presented, along with the corresponding *P* value in parentheses. (**D**) Correlation between RSK1 and MCM8 protein expression in 12 GBM cells lines from the CCLE. Pearson correlation coefficient is presented, along with the *P* value in parentheses. Before analysis, all proteomic data were transformed by 2^x.

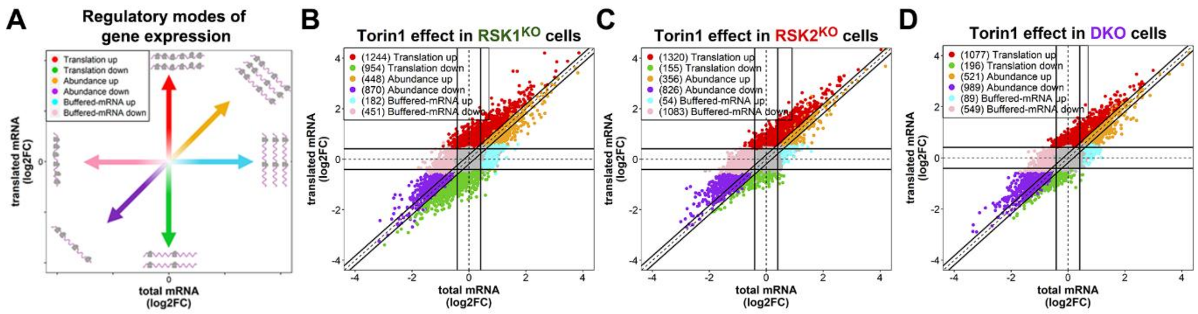

Figure S12. Effect of Torin1 on translation efficiency (TE) in RSK knockout LN18^{CRISPR} cells. (**A**) Schematic representation of the regulatory modules of gene expression based on changes in the transcriptome (total mRNA) and translatome (translated mRNA).[17] Created in BioRender. Roffe, M. (2025) https://BioRender.com/7mhm9os. (**B-D**) Graphs showing changes in gene expression and the regulatory modes assigned according to the anota2seq method for Torin1-treated (**B**) RSK1^{KO}, (**D**) RSK2^{KO}, and (**H**) DKO cells. The number of genes classified in each mode is indicated within parentheses.

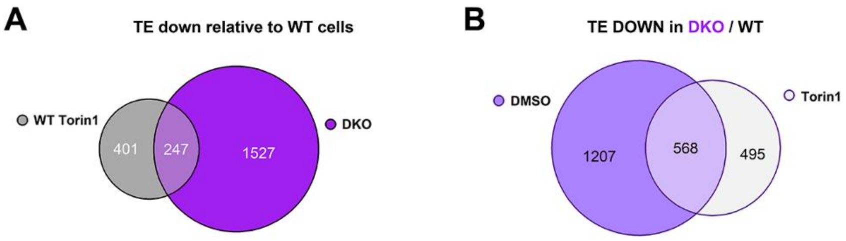

Figure S13. Effect of RSK isoforms loss on TE in DKO LN18^{CRISPR} cells. (**A**) Venn-diagram showing the number of mRNAs that were downregulated by TE in DKO cells relative to WT cells, and in Torin1-treated WT cells. (**B**) Venn-diagram showing the number of mRNAs that were downregulated in DKO cells relative to WT cells, in the absence (DMSO) and presence of Torin1.

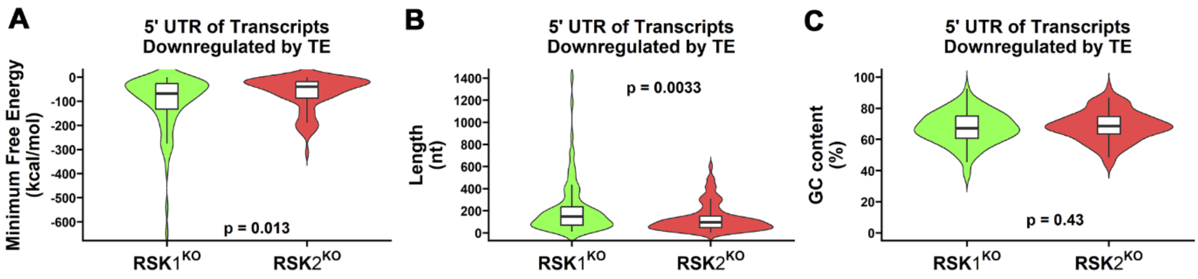

Figure S14. Analysis of the 5′ UTRs of transcripts downregulated in RSK1^{KO} and RKS2^{KO} LN18^{CRISPR} cells. (**A**) Minimum free energy was predicted for the 5′ UTRs of the 92 most downregulated and unique transcripts of RSK1^{KO} and RSK2^{KO} cells using the RNAfold algorithm from the ViennaRNA package.[18] (**B**) The length of the 5′ UTRs was calculated for the same set of transcripts. (**C**) GC content was calculated for the 5′ UTRs of these transcripts. P-values from Wilcoxon rank-sum tests are shown in the graphs.

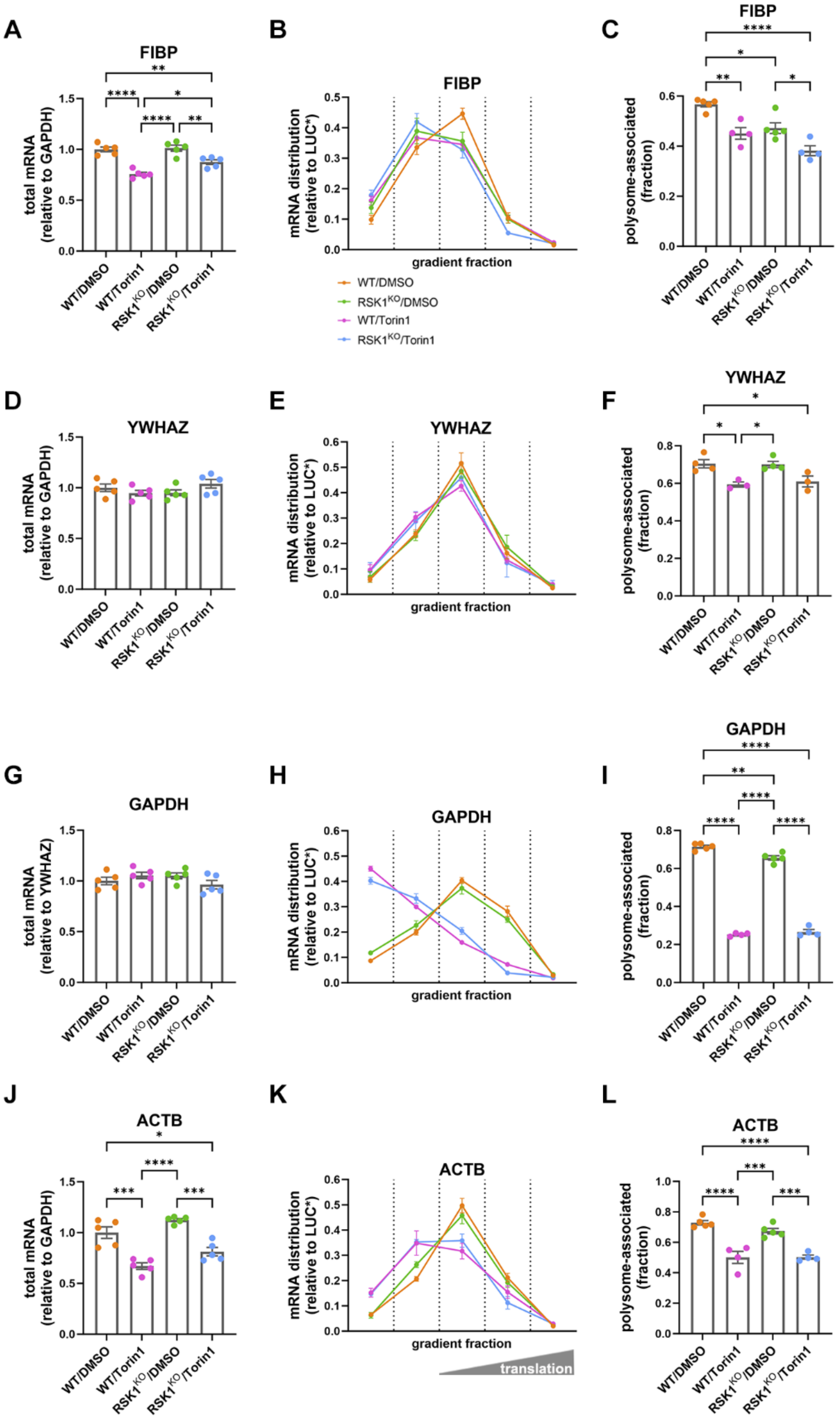

Figure S15. Total mRNA and its distribution across traditional polysome profiles for FIBP, YWHAZ, GAPDH and ACTB. (**A**) Graph showing total FIBP mRNA levels. (**B**) Distribution of FIBP mRNA in the polysome profile of Fig. 5I. (**C**) Graph showing the fraction of FIBP mRNA associated with ≥ 3n polysomes. (**D**) Graph showing total YWHAZ mRNA levels. (**E**) Distribution of YWHAZ

mRNA in the polysome profile. (**F**) Graph showing the fraction of YWHAZ mRNA associated with ≥ 3n polysomes. (**G**) Graph showing total GAPDH mRNA levels. (**H**) Distribution of GAPDH mRNA in the polysome profile. (**I**) Graph showing the fraction of GAPDH mRNA associated with ≥ 3n polysomes. (**J**) Graph showing total β-actin mRNA levels. (**K**) Distribution of β-actin mRNA in the polysome profile. (**L**) Graph showing the fraction of β-actin mRNA associated with ≥ 3n polysomes. The mean values of at least three independent experiments (±SEM) are presented. *$P < 0.05$ (one-way ANOVA followed by Tukey's post hoc test).

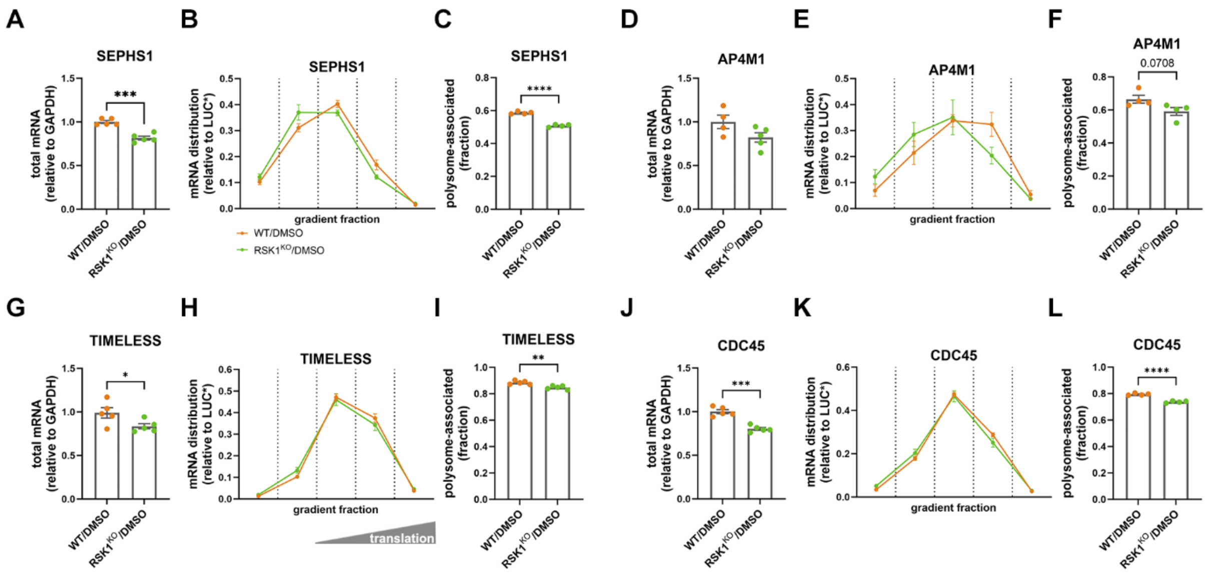

Figure S16. Distribution of mRNAs downregulated in RSK1^{KO} cells across traditional polysome profiles of RSK1^{KO} and WT LN18^{CRISPR} cells. mRNAs classified as downregulated by TE in the translatomics, were analyzed in traditional polysome profiles (Fig. 5I). (**A**) Graph showing total SEPHS1 mRNA levels. (**B**) Distribution of SEPHS1 mRNA in the polysome profile. (**C**) Graph showing the fraction of SEPHS1 mRNA associated with ≥ 3n polysomes. (**D**) Graph showing total AP4M1 mRNA levels. (**E**) Distribution of AP4M1 mRNA in the polysome profile. (**F**) Graph showing the fraction of AP4M1 mRNA associated with ≥ 3n polysomes. (**G**) Graph showing total TIMELESS mRNA levels. (**H**) Distribution of TIMELESS mRNA in the polysome profile. (**I**) Graph showing the fraction of TIMELESS mRNA associated with ≥ 3n polysomes. (**J**) Graph showing total CDC45 mRNA levels. (**K**) Distribution of CDC45 mRNA in the polysome profile. (**L**) Graph showing the fraction of CDC45 mRNA associated with ≥ 3n polysomes. The mean values of at least three independent experiments (±SEM) are presented. *$P < 0.05$ (Student's t-test).

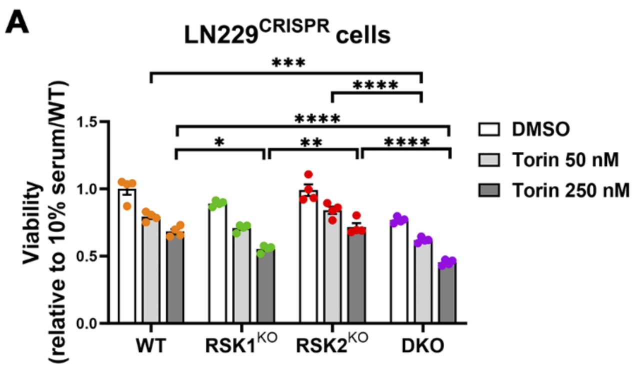

Figure S17. Viability of LN229^{CRISPR} cells in the presence of Torin1. (**A**) Cell viability was measured for LN229^{CRISPR} cells after the addition of DMSO or the indicated concentrations of Torin1 for 24 hours. The mean values of four independent experiments (±SEM) are presented.

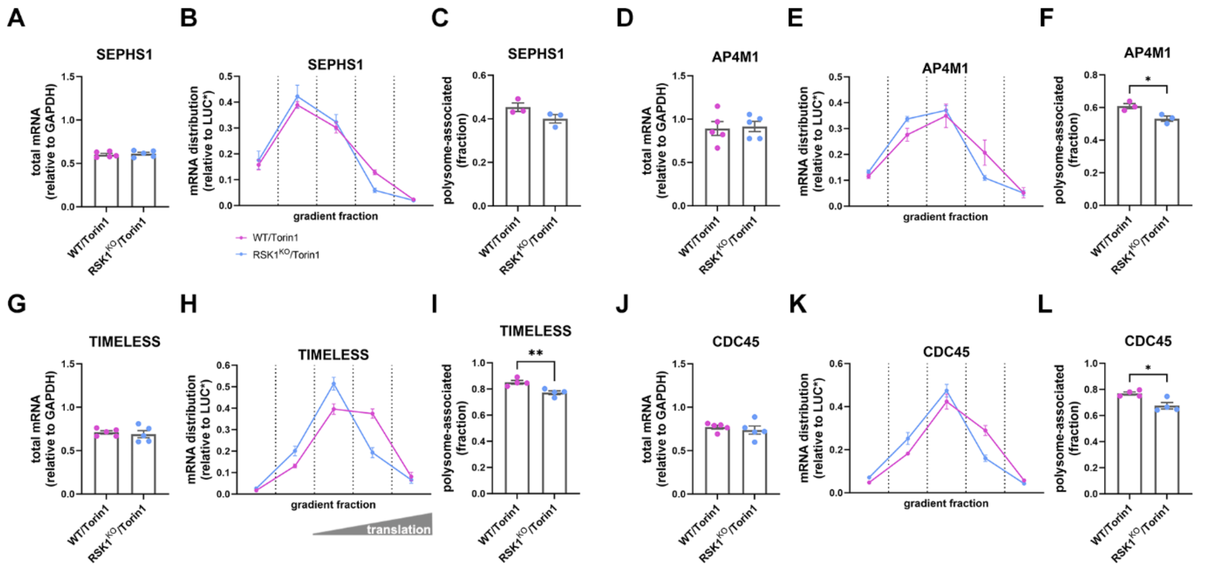

Figure S18. Distribution of mRNAs in traditional polysome profiles of RSK1^{KO} and WT LN18^{CRISPR} cells in the presence of Torin1. mRNAs classified as downregulated by TE in the translatomics, were analyzed in traditional polysome profiles of Torin1-treated cells (Fig. 5I). (**A**) Graph showing total SEPHS1 mRNA levels. (**B**) Distribution of SEPHS1 mRNA in the polysome profile. (**C**) Graph showing the fraction of SEPHS1 mRNA associated with ≥ 3n polysomes. (**D**) Graph showing total AP4M1 mRNA levels. (**E**) Distribution of AP4M1 mRNA in the polysome profile. (**F**) Graph showing the fraction of AP4M1 mRNA associated with ≥ 3n polysomes. (**G**) Graph showing total TIMELESS mRNA levels. (**H**) Distribution of TIMELESS mRNA in the polysome profile. (**I**) Graph showing the fraction of TIMELESS mRNA associated with ≥ 3n polysomes. (**J**) Graph showing total CDC45 mRNA levels. (**K**) Distribution of CDC45 mRNA in the polysome profile. (**L**) Graph showing the fraction of CDC45 mRNA associated with ≥ 3n polysomes. The mean values of at least three independent experiments (±SEM) are presented. *$P < 0.05$ (Student's t-test).

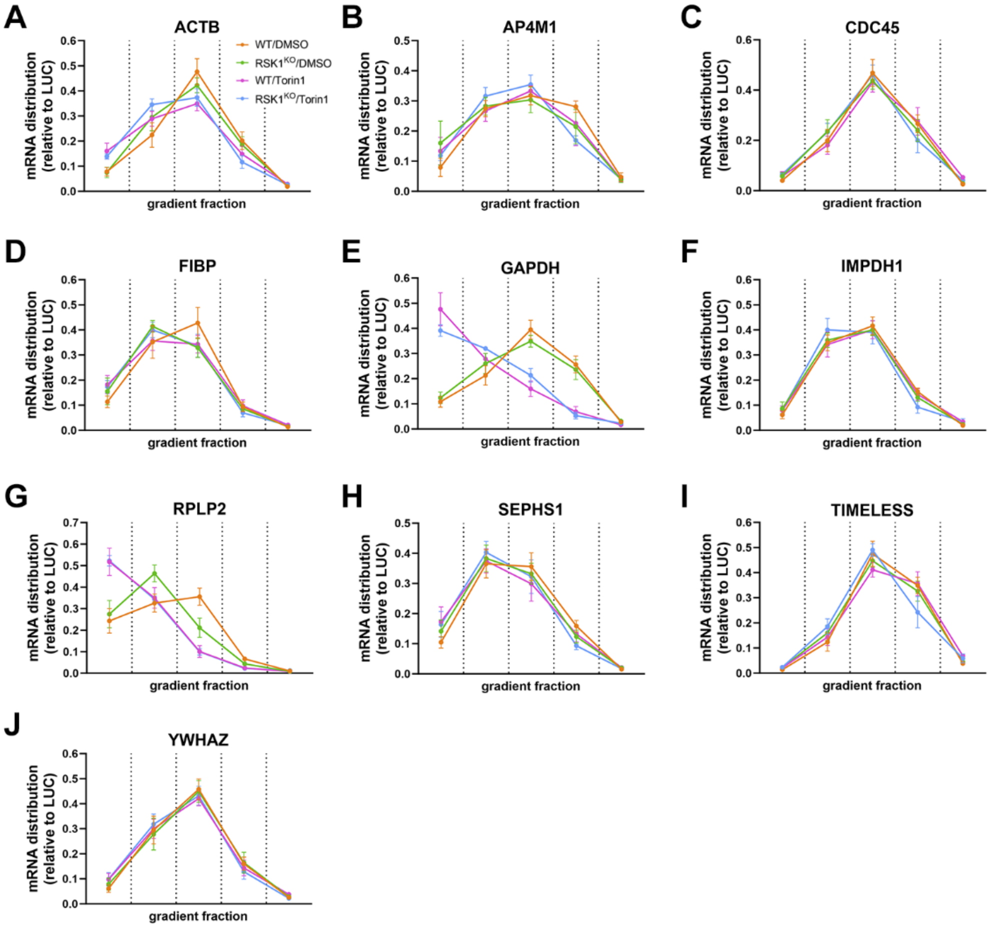

Figure S19. Distribution of mRNAs in traditional polysome profiles of RSK1^{KO} and WT LN18^{CRISPR} cells before correction. Graphs for (**A**) β-actin, (**B**) AP4M1, (**C**) CDC45, (**D**) FIBP, (**E**) GAPDH, (**F**) IMPDH1, (**G**) RPLP2, (**H**) SEPHS1, (**I**) TIMELESS, and (**J**) YWHAZ. The mean values of at least three independent experiments (±SEM) are presented.

SUPPLEMENTARY FILES LEGENDS

File S1. Excel file containing the mRNAs downregulated in the translatome of $RSK1^{KO}$, $RSK2^{KO}$, and DKO cells relative to WT $LN18^{CRISPR}$ cells.

File S2. Excel file containing the Biological Processes downregulated in the translatome of $RSK1^{KO}$, $RSK2^{KO}$, DKO cells relative to WT $LN18^{CRISPR}$ cells.

File S3. Excel file containing the RSK1 and RSK2 dependent mRNAs that were selected after applying stringent-filters. For the proteins coded by those mRNAs is also presented their correlation with RSK1 calculated from a quantitative proteomics analysis of 12 GBM cell lines from the CCLE.

File S4. Excel file containing the mRNAs downregulated by TE in $RSK1^{KO}$, $RSK2^{KO}$, and DKO cells relative to WT $LN18^{CRISPR}$ cells.

File S5. Excel file containing the mRNAs downregulated by TE after treatment with Torin1 of WT, $RSK1^{KO}$, $RSK2^{KO}$ and DKO $LN18^{CRISPR}$ cells.

File S6. Excel file containing the mRNAs downregulated by TE in $RSK1^{KO}$, $RSK2^{KO}$, DKO cells relative to WT $LN18^{CRISPR}$ cells in the presence of Torin1.

File S7. Excel file containing the Biological Processes downregulated by TE in $RSK1^{KO}$ relative to WT $LN18^{CRISPR}$ cells in the presence of Torin1.

File S8. Excel file containing the mRNAs downregulated in the transcriptome of $RSK1^{KO}$ and $RSK2^{KO}$ cells relative to WT $LN18^{CRISPR}$ cells, and the comparison with the transcriptome of $RSK1^{KO}$ and $RSK2^{KO}$ U251MG glioblastoma cells from Yang et al.[8]

SUPPLEMENTARY REFERENCES

1. Ray-David H, Romeo Y, Lavoie G, et al. RSK promotes G2 DNA damage checkpoint silencing and participates in melanoma chemoresistance. *Oncogene*. 2013;32(38):4480-4489. doi:10.1038/onc.2012.472

2. Roffé M, Hajj GNM, Azevedo HF, Alves VS, Castilho BA. IMPACT is a developmentally regulated protein in neurons that opposes the eukaryotic initiation factor 2α kinase GCN2 in the modulation of neurite outgrowth. *J Biol Chem*. 2013;288(15):10860-10869. doi:10.1074/jbc.M113.461970

3. Ran FA, Hsu PD, Wright J, Agarwala V, Scott DA, Zhang F. Genome engineering using the CRISPR-Cas9 system. *Nat Protoc*. 2013;8(11):2281-2308. doi:10.1038/nprot.2013.143

4. Liang S, Bellato HM, Lorent J, et al. Polysome-profiling in small tissue samples. *Nucleic Acids Res*. 2018;46(1):e3. doi:10.1093/nar/gkx940

5. Langfelder P, Horvath S. WGCNA: an R package for weighted correlation network analysis. *BMC Bioinformatics*. 2008;9(1):559. doi:10.1186/1471-2105-9-559

6. Oertlin C, Lorent J, Murie C, Furic L, Topisirovic I, Larsson O. Generally applicable transcriptome-wide analysis of translation using anota2seq. *Nucleic Acids Res*. 2019;47(12):e70. doi:10.1093/nar/gkz223

7. Gandin V, Sikström K, Alain T, et al. Polysome Fractionation and Analysis of Mammalian Translatomes on a Genome-wide Scale. *JoVE (Journal of Visualized Experiments)*. 2014;87(87):e51455. doi:10.3791/51455

8. Yang WS, Caliva MJ, Khadka VS, et al. RSK1 and RSK2 serine/threonine kinases regulate different transcription programs in cancer. *Front Cell Dev Biol*. 2022;10:1015665. doi:10.3389/fcell.2022.1015665

9. Roux PP, Ballif BA, Anjum R, Gygi SP, Blenis J. Tumor-promoting phorbol esters and activated Ras inactivate the tuberous sclerosis tumor suppressor complex via p90 ribosomal S6 kinase. *Proc Natl Acad Sci U S A*. 2004;101(37):13489-13494. doi:10.1073/pnas.0405659101

10. Carrière A, Cargnello M, Julien LA, et al. Oncogenic MAPK signaling stimulates mTORC1 activity by promoting RSK-mediated raptor phosphorylation. *Curr Biol*. 2008;18(17):1269-1277. doi:10.1016/j.cub.2008.07.078

11. Thoreen CC, Kang SA, Chang JW, et al. An ATP-competitive mammalian target of rapamycin inhibitor reveals rapamycin-resistant functions of mTORC1. *J Biol Chem*. 2009;284(12):8023-8032. doi:10.1074/jbc.M900301200

12. Shahbazian D, Roux PP, Mieulet V, et al. The mTOR/PI3K and MAPK pathways converge on eIF4B to control its phosphorylation and activity. *EMBO J*. 2006;25(12):2781-2791. doi:10.1038/sj.emboj.7601166

13. Roux PP, Shahbazian D, Vu H, et al. RAS/ERK signaling promotes site-specific ribosomal protein S6 phosphorylation via RSK and stimulates cap-dependent translation. *J Biol Chem*. 2007;282(19):14056-14064. doi:10.1074/jbc.M700906200

14. Wang X, Li W, Williams M, Terada N, Alessi DR, Proud CG. Regulation of elongation factor 2 kinase by p90(RSK1) and p70 S6 kinase. *EMBO J*. 2001;20(16):4370-4379. doi:10.1093/emboj/20.16.4370

15. Zhao Y, Bjorbaek C, Moller DE. Regulation and interaction of pp90(rsk) isoforms with mitogen-activated protein kinases. *J Biol Chem*. 1996;271(47):29773-29779. doi:10.1074/jbc.271.47.29773

16. Nusinow DP, Szpyt J, Ghandi M, et al. Quantitative Proteomics of the Cancer Cell Line Encyclopedia. *Cell*. 2020;180(2):387-402.e16. doi:10.1016/j.cell.2019.12.023

17. Hajj GNM, Nunes PBC, Roffe M. Genome-wide translation patterns in gliomas: An integrative view. *Cell Signal*. 2021;79:109883. doi:10.1016/j.cellsig.2020.109883

18. Lorenz R, Bernhart SH, Höner zu Siederdissen C, et al. ViennaRNA Package 2.0. *Algorithms for Molecular Biology*. 2011;6(1):1-14. doi:10.1186/1748-7188-6-26/TABLES/2
